# A Component of the Xanthomonadaceae Type IV Secretion System Combines a VirB7 Motif with a N0 Domain Found in Outer Membrane Transport Proteins

**DOI:** 10.1371/journal.ppat.1002031

**Published:** 2011-05-12

**Authors:** Diorge P. Souza, Maxuel O. Andrade, Cristina E. Alvarez-Martinez, Guilherme M. Arantes, Chuck S. Farah, Roberto K. Salinas

**Affiliations:** Department of Biochemistry, Institute of Chemistry, University of São Paulo, São Paulo, Brazil; The Rockefeller University, United States of America

## Abstract

Type IV secretion systems (T4SS) are used by Gram-negative bacteria to translocate protein and DNA substrates across the cell envelope and into target cells. Translocation across the outer membrane is achieved via a ringed tetradecameric outer membrane complex made up of a small VirB7 lipoprotein (normally 30 to 45 residues in the mature form) and the C-terminal domains of the VirB9 and VirB10 subunits. Several species from the genera of *Xanthomonas* phytopathogens possess an uncharacterized type IV secretion system with some distinguishing features, one of which is an unusually large VirB7 subunit (118 residues in the mature form). Here, we report the NMR and 1.0 Å X-ray structures of the VirB7 subunit from *Xanthomonas citri* subsp. citri (VirB7_XAC2622_) and its interaction with VirB9. NMR solution studies show that residues 27–41 of the disordered flexible N-terminal region of VirB7_XAC2622_ interact specifically with the VirB9 C-terminal domain, resulting in a significant reduction in the conformational freedom of both regions. VirB7_XAC2622_ has a unique C-terminal domain whose topology is strikingly similar to that of N0 domains found in proteins from different systems involved in transport across the bacterial outer membrane. We show that VirB7_XAC2622_ oligomerizes through interactions involving conserved residues in the N0 domain and residues 42–49 within the flexible N-terminal region and that these homotropic interactions can persist in the presence of heterotropic interactions with VirB9. Finally, we propose that VirB7_XAC2622_ oligomerization is compatible with the core complex structure in a manner such that the N0 domains form an extra layer on the perimeter of the tetradecameric ring.

## Author Summary

Many aspects of bacterial life require that they translocate proteins to the cell exterior. To do this, different macromolecular secretion systems of varying complexity have evolved (Type I–VI secretion systems). These secretion systems are often at the front lines of pathogen-host interactions and are important for the development of disease. In this work, we have determined the structure and studied the interactions of an unusually large VirB7 subunit (VirB7_XAC2622_) of the outer membrane pore of the Type IV secretion system found in the *Xanthomonas* genera of phytopathogens. Its mosaic structure combines a canonical VirB7 N-terminal region with a C-terminal globular domain whose topology is observed in a relatively limited set of proteins, all involved in molecular transport across outer membranes. Our results lead to the hypothesis that the VirB7_XAC2622_ globular domains can form an extra ring around the perimeter of the outer membrane pore and reveal deeper structural and evolutionary relationships among bacterial macromolecular secretion systems that have evolved to adopt a variety of functions, including structural modules in outer membrane pores (secretins from Type II, III and IV secretion systems, Type IV pili and filamentous phages), signal-transduction modules in TonB-dependent receptors and membrane-penetrating devices in T6SS and long-tailed bacteriophages.

## Introduction

Bacteria employ multiprotein secretion systems to translocate effector proteins or nucleoprotein complexes across the cell envelope where they modulate the bacterium's interactions with the environment. In Gram-negative bacteria, the secretion systems have been classified into 6 different types [1], [2]. The type IV secretion systems (T4SSs) are ancestrally related to bacterial conjugation machines [3] and are able to translocate proteins and/or protein-DNA complexes to the extracellular milieu or the host interior, in many cases contributing to the ability of the bacterial pathogen to colonize the host and evade its immune system [4]. T4SSs are important in the pathogenic mechanism of many microbes, including the animal pathogens *Legionella pneumophila* (Legionnaires' disease; [5]), *Bordetella pertussis* (whooping cough; [6]), *Coxiella burnetii* (Q fever; [7]), *Bartonella henselae* (cat scratch disease, [8]), *Brucella* spp. (brucellosis; [9]) and *Helicobacter pylori* (gastritis, ulcers, stomach cancer; [10]), as well as the plant pathogen *Agrobacterium tumefaciens* (crown gall disease; [11]) which has the prototype T4SS, composed of 12 proteins, VirB1-VirB11 and VirD4 [12].

*Xanthomonas citri* subsp. citri (formerly *Xanthomonas axonopodis* pv. citri) (Xac) is a gammaproteobacterial phytopathogen which causes citrus canker, a disease that affects all citrus plants and has significant economic impact worldwide [13]. The Xac genome was sequenced and many potential virulence-related genes were identified [14], including an 18 kb chromosomal *vir* locus that codes for a T4SS [13], [14], [15]. Orthologous T4SSs have been identified in the closely related species *Xanthomonas campestris* pv. campestris (strains ATCC 33913 [14], 8004 [16] and B100 [17]), *Xanthomonas albilineans* [18], *Xanthomonas campestris* pv. vasculorum NCPPB702 [19], *Xanthomonas campestris* pv. musacearum NCPPB4381 [19] and *Stenotrophomonas maltophilia* (strains K279A [20] and R551-3 [21]). The *vir* locus is incomplete in *Xanthomonas campestris* pv. vesicatoria [22] and is absent in four *Xanthomonas oryzae* strains (KACC10331 [23], MAFF311018 [24], PXO99A [25] and BLS256 (GenBank database AAQN00000000)) and in two *Xanthomonas* fuscans subsp. *aurantifolii* lineages (10535 and 11122 [26]). The unrelated Gram-negative bacteria *Neisseria flavescens* SK114 (GenBank database ACQV00000000) and *Neisseria* sp. oral taxon 014 str. F0314 (GenBank database ADEA00000000) also present a locus highly similar to the T4SS loci found in the Xanthomonadaceae family. Some *Xanthomonas* genomes, including Xac, carry megaplasmids that code for a second T4SS probably involved in plasmid mobilization and whose structural components exhibit only very low sequence identity to their counterparts in the chromosomally encoded T4SS under study [15], [27].

One particularly distinctive feature of the Xanthomonad T4SSs is related to the VirB7 component. In well-characterized T4SSs, VirB7 is a lipoprotein attached to the periplasmic side of the outer membrane [28]. It has been shown to interact with several T4SS subunits [29], including itself [30] and the VirB9 C-terminal region [31], [32]. The structure of a VirB7-VirB9-VirB10 complex (TraN-TraO-TraF) from the T4SS of the conjugative plasmid pKM101 demonstrated that these proteins form a hetero-tetradecameric outer membrane channel through which the substrates pass [33], [34]. Structures of the complex formed between the TraO C-terminal domain and TraN revealed that the 33-residue TraN forms an extended structure that winds around the TraO β-sandwich [34], [35].

Members of the VirB7 family are typically 45–65 residues long [36], becoming 15–20 residues shorter after removal of the N-terminal signal sequence and covalent attachment to lipid molecules. In Xac, several lines of evidence indicate that the gene *xac2622* codes for a VirB7 equivalent; specifically, the gene position, the presence of signals for outer membrane localization and lipidation and the interaction of its product with VirB9 [15]. However, XAC2622 does not exhibit any sequence similarity with the VirB7 family and it is much larger than a typical VirB7 protein (139 and 118 residues before and after signal sequence removal, respectively).

In order to understand the unique structural features of Xanthomonad VirB7, we have solved the solution nuclear magnetic resonance (NMR) and X-ray crystal structures of XAC2622, which we now call VirB7_XAC2622_, and studied its interaction with VirB9. VirB7_XAC2622_ presents all of the characteristics of VirB7: predicted signal peptide and lipobox sequences followed by a short and extended region that contains the VirB9 binding site. However, unlike other VirB7 proteins, VirB7_XAC2622_ has an extra domain whose topology is strikingly similar to that of N0 domains found in proteins from different multi-subunit complexes involved in transport across the bacterial outer membrane. We show that VirB7_XAC2622_ oligomerizes through interactions involving conserved residues in the folded domain and the unfolded N-terminus and that oligomerization and the formation of the VirB7_XAC2622_-VirB9 complex can occur simultaneously. We propose that these interactions are compatible with the tetradecameric core complex structure, resulting in the formation of an extra ring layer.

## Results

### Solution Structure of Xac VirB7 (VirB7_XAC2622_24–139_)

The construct used for NMR structure determination corresponds to residues 24–139 of VirB7_XAC2622_ preceded by a Gly-Ser-His-Met sequence. Bioinformatics analysis predicts that residues 1–21 would be removed and Cys22 lipidated, leading to anchoring in the bacterial outer membrane [15]. Approximately 98% of main chain and aliphatic side chain and 93% of aromatic side chain ^1^H, ^13^C and ^15^N resonances were assigned (Table 1 and Figure S1). A total of 2176 experimental NMR restraints, consisting of 2056 interproton distances and 120 torsion angles, were used in the structure calculation (Table 1). A small set of nuclear Overhauser effect (NOE) cross-peaks that were not assigned by CYANA [37] were subsequently identified as resulting from intermolecular contacts (see below).

**Table 1:** Assignment and structural statistics for the solution structure of VirB7_XAC2622_24–139_.

Completeness of resonance assignments (%)^a^	97.6
Backbone	98.4
Side chain	97.6
Aromatic	93.5
Total unambiguous distance constraints	2056
Intra-residue (i = j)	438
Sequential (|i−j| = 1)	573
Medium range (1<|i−j|<5)	382
Long range (|i−j|≥5)	663
Total dihedral angle constraints	120
Phi/Psi	60/60
Average CYANA target function value (Å^2^)	0.73±0.10
Residue constraints violations	
NOE violations/structure (>0.3 Å)	0.05±0.022
NOE violations/structure (>0.5 Å)	0
RMSD from experimental distance restraints (Å)	0.013±0.001
Dihedral angle violations/structure (>5 deg.)	0.40±0.58
RMSD from experimental dihedral restraints (deg.)	0.64±0.11
RMSD from ideal geometry	
Bond lengths (Å)	0.0032±0.0001
Bond angles (deg.)	0.398±0.008
Improper angles (deg.)	0.357±0.014
Average pairwise RMSD (Å)^b^	
Backbone atoms	0.47±0.10
Heavy atoms	0.92±0.09
Ramachandran plot statistics (%)	
Most favored regions	84.9
Additional allowed regions	14.9
Generously allowed	0.0
Disallowed regions	0.2^c^
Residual dipolar couplings^d^	
^1^H-^15^N	35
Average Q factor with respect to solution structure	0.271±0.019
Q factor with respect to crystal structure	0.187

^*a*^The GSHM coded by the N-terminal tag was removed for the analysis performed by the AVS program [89].

^*b*^RMSD calculated for residues 52–133.

^*c*^Residue A27 in two structures, K25 and A123 in one conformer (all in different conformers).

^*d*^RDCs were not used in the structure calculation.

**Figure 1:**
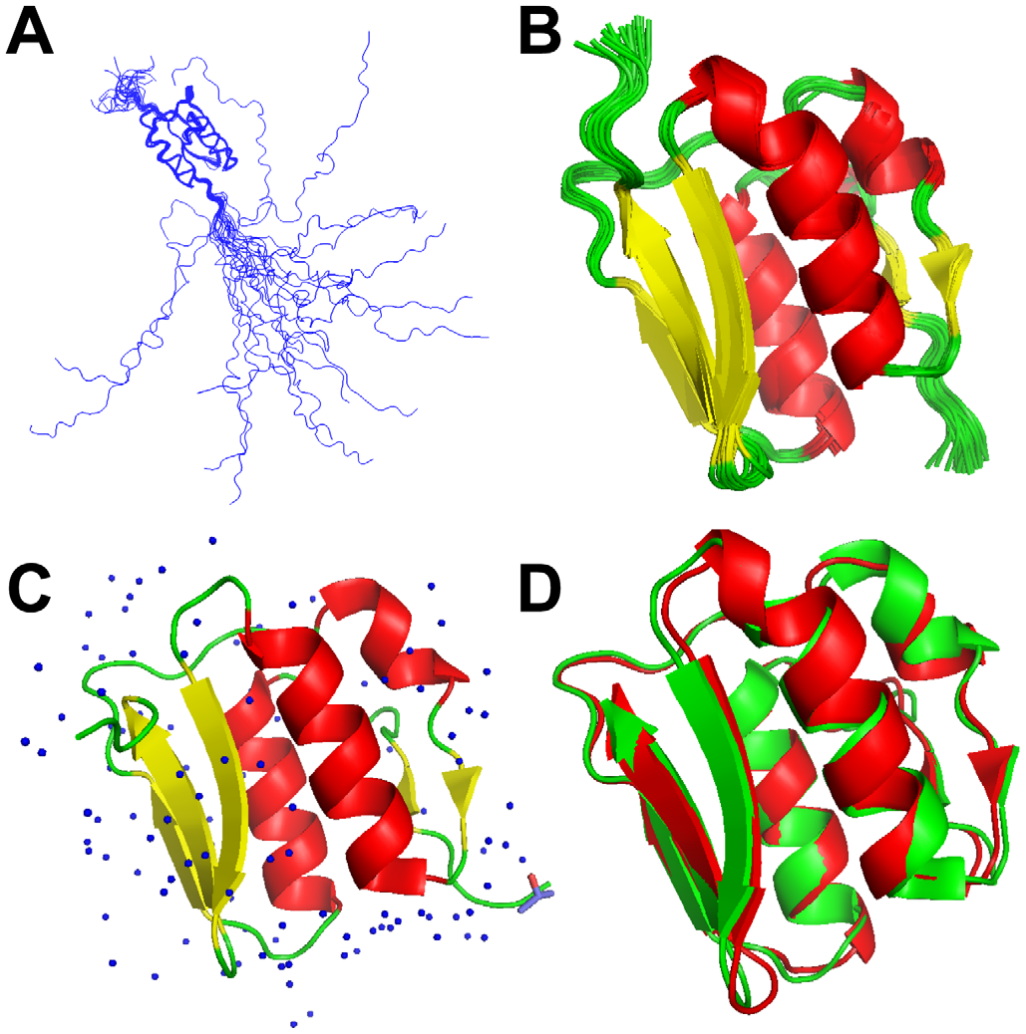
NMR and X-ray models of VirB7_XAC2622_. Superposition of 20 lowest energy VirB7_XAC2622_24–139_ solution structures: (A) backbone traces of the full-length protein (residues 24–139) and (B) ribbon representation of the folded domain (residues 51–134) in the NMR ensemble. (C) Ribbon representation of the X-ray crystal structure of VirB7_XAC2622_51–134_. Water molecules and one isopropanol ligand are depicted. (D) Superposition of the X-ray (green) and NMR (lowest energy model; red) structures of the VirB7_XAC2622_ globular domain.

The solution structure of VirB7_XAC2622_24–139_ consists of a globular domain (residues 52–133), flanked by a long disordered N-terminus (amino acids 24–51) and a short flexible C-terminus (residues 134–139) (Figure 1A). The globular domain is composed of two α-helices sandwiched between a mixed three stranded β-sheet on one side and an antiparallel two-stranded β-sheet plus a short 3_10_ helix on the other (Figure 1B). The NMR structures were independently validated by ^1^H-^15^N residual dipolar couplings (RDCs). The average Q factor obtained by fitting RDCs from secondary structure elements to the whole NMR ensemble is 0.271±0.019, consistent with a good quality structure [38], [39], [40].

### High Resolution X-ray Crystal Structure of the VirB7_XAC2622_ Globular Domain

To better characterize the globular domain, we cloned and purified a recombinant fragment encompassing residues 51–134 (VirB7_XAC2622_51–134_) which was submitted to crystallization trials that resulted in large plates (Figure S2) which belong to space group C222_1_ and diffracted up to 1.0 Å. Molecular replacement was performed using the solution structure of the VirB7_XAC2622_ globular domain as the search model. Details of the data collection and structure refinement are listed in Table 2 and the model is presented in Figure 1C. There was clear electron density for all residues, with the exception of the first amino acid (threonine 51) and residues 122 and 123 in the loop between β4 and β5. As expected, the crystal structure is very similar to the NMR ensemble (Figure 1D), displaying an average root mean square deviation (RMSD) for the backbone heavy atoms and all heavy atoms of 1.05±0.05 Å and 1.61±0.06 Å for residues 53–130, and excellent agreement with the backbone ^1^H-^15^N RDCs [Q factor of 0.187 (regular secondary structures only) and 0.272 (all residues)].

**Table 2:** Crystallographic data collection and refinement statistics of VirB7_XAC2622_51–134_.

Data Collection	
Wavelength (Å)	0.9537
Space group	C222_1_
Cell axis *a*, *b*, *c* (Å)	27.76, 55.81, 83.10
Resolution (Å)	30.00 – 1.04 (1.08 – 1.04)^a^
No. of observations	367270
Unique reflections	30386
*R*_merge_ b	0.068 (0.293)^a^
〈*I*/*σ*(*I*)〉	34.8 (5.1)^a^
Completeness (%)	96.3 (79.0)^a^
Redundancy	12.1 (8.9)^a^

Refinement Statistics	
Protein molecules per ASU	1
Amino acid residues per ASU	84
Total non-hydrogen atoms	738
Protein atoms	619
Water molecules	115
Ligand atoms	4
*R*_work_/*R*_free_ c	0.130/0.152 (0.191/0.209)^a^
Figure of Merit	0.92
Average *B*-factor (Å^2^)	
Protein all atoms	10.4
Protein main chain	7.8
Protein side chain	12.6
Water	23.6
Ligand	17.2
RMSD bond lengths (Å)	0.013
RMSD bond angles (deg.)	1.690
Ramachandran plot statistics (%)	
Most favored regions	95.1
Additionally allowed regions	4.9

^*a*^Numbers in parentheses represent data for the highest resolution shell.

^*b*^ *R*_merge_ = Σ*_hkl_* Σ*_i_* |*I_i_*(*hkl*)−〈I(*hkl*)〉 |/Σ*_hkl_* Σ*_i_I_i_*(*hkl*).

^*c*^ *R*_work_ = Σ|F_obs_−F_calc_|/ΣF_obs_. The *R*_free_ value is the same as *R*_work_ but calculated on 5% of the data not included in the refinement. ASU, asymmetric unit.

### VirB7 Oligomerization

**Figure 2:**
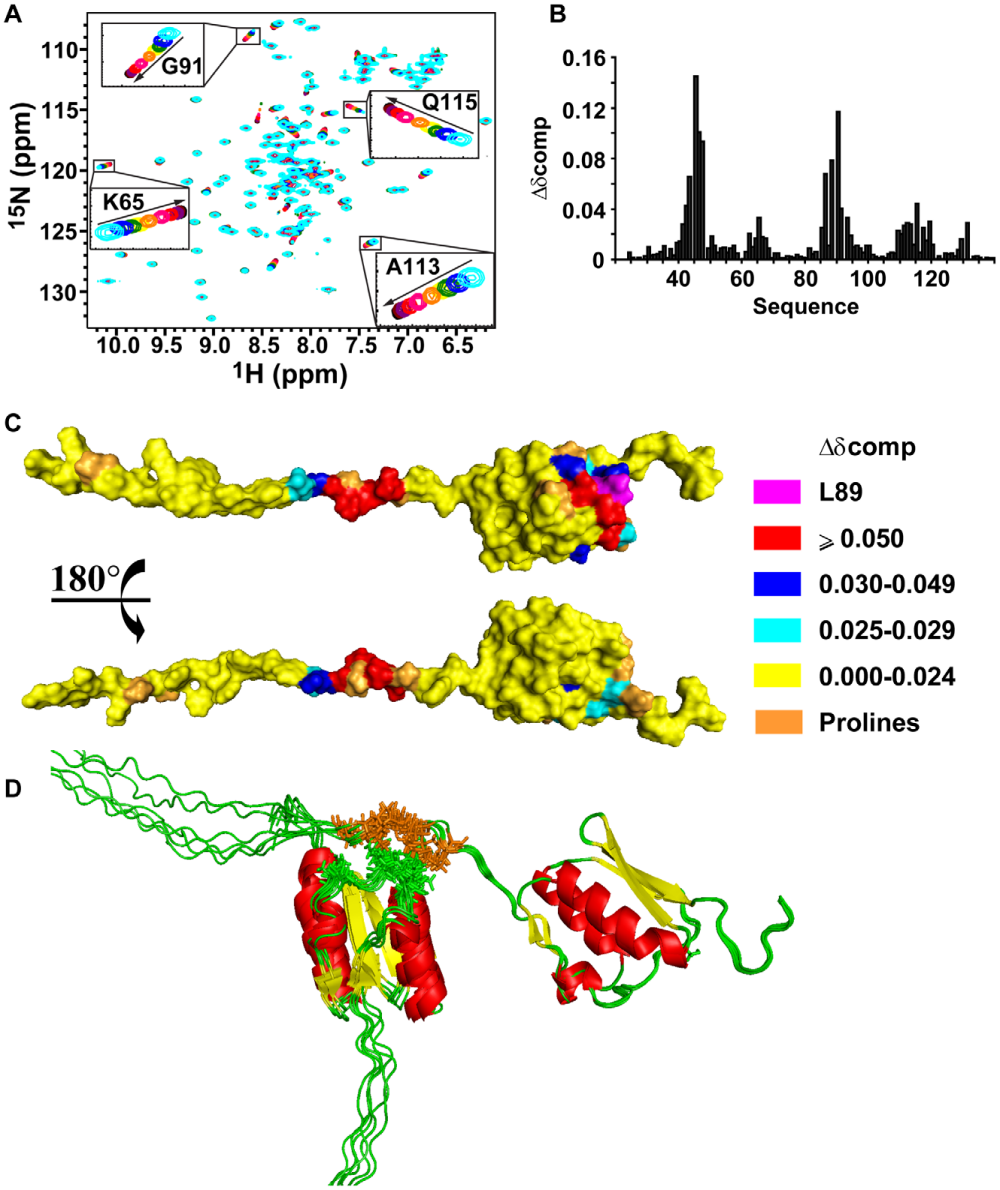
VirB7_XAC2622_24–139_ oligomerization. (A) Superposition of ^15^N-HSQC spectra of ^15^N-VirB7_XAC2622_24–139_ at different concentrations. Cyan: 850 µM; blue: 600 µM; green: 400 µM; yellow: 300 µM; orange: 200 µM; pink: 100 µM; red: 50 µM; purple: 25 µM; brown: 13 µM and black: 7 µM. The inserts are amplifications of selected spectral regions showing peak movements as the protein concentration varies from 850 to 7 µM in the direction of the arrows. (B) Weighted chemical shift changes (Δδcomp) observed upon dilution from 100 µM to 7 µM. (C) Surface representation of VirB7_XAC2622_24–139_ colored according to the weighted chemical shift changes. Residues involved in oligomerization are located in the folded domain and in the unfolded N-terminus. ^1^H-^15^N correlation of Leucine 89 (L89) was not detected in any ^15^N-HSQC spectrum probably due to chemical exchange. (D) Ribbon representations of docking models of the VirB7_XAC2622_ – VirB7_XAC2622_ interaction. Helices, β-strands and coil regions are colored red, yellow and green, respectively. Residues involved in intermolecular NOEs are shown as stick models colored brown (residues A43, T45, E46, I47, L49) and green (T63, S85, D86, Y87, T88, I90). Six models are shown.

Changes in ^15^N heteronuclear single-quantum coherence (^15^N-HSQC) cross-peak positions as a function of protein concentration indicated that VirB7_XAC2622_ oligomerizes in fast exchange on the NMR time scale (Figure 2A). Consistent with this observation, alterations in ^15^N T_1_ and T_2_ relaxation times indicated that the overall tumbling slows significantly at higher protein concentrations (see below). Chemical shift perturbation data showed that two regions are involved in VirB7_XAC2622_ self-interactions: a region in the unfolded N-terminus (residues 42–49) and a patch on the surface of the globular domain made up of residues 63–65, 85–93, 111–119 and 131 (Figure 2B and C).

Two possible interaction schemes can be envisaged that would be compatible with the chemical shift perturbation data: i) a side-by-side arrangement in which the N-terminal unfolded segment and the globular domain of one molecule interact, respectively, with the same regions of a second molecule, or ii) a head-to-tail complex arrangement where the N-terminus of one molecule recognizes the folded domain of the other. In order to test the hypothesis of the formation of a head to tail complex, we performed a titration of the ^15^N-VirB7_XAC2622_51–134_ globular domain with an unlabeled peptide encompassing residues 38–52 from the N-terminal region (VirB7_XAC2622_38–52_). During the titration, we observed changes in the same cross-peaks which were perturbed in the entire protein in a concentration dependent manner (Figure S3). This hypothesis was corroborated by the analysis of a set of 13 NOEs not assigned by CYANA [37], which showed that they could be accounted for by the formation of head-to-tail dimers. Indeed, those NOEs were observed between proton pairs derived from amino acids whose main-chain ^1^H-^15^N chemical shifts are perturbed in a concentration dependent manner (the NOEs are listed in the Materials and Methods).

We then used the set of 13 intermolecular NOEs as geometric restraints to drive computational docking simulations of the VirB7_XAC2622_ dimer using HADDOCK2.0 [41]. In one simulation, a fragment corresponding to the disordered N-terminal region (residues 24–50) was docked with a fragment corresponding to the globular domain (residues 51–139) while the second simulation involved the docking between two full length proteins (residues 24–139). The resulting models from both simulations were highly similar. In the second simulation three of the thirteen NOEs were violated in all solutions by approximately 1.4 to 3 Å. They correspond to weak peaks and could have contributions of spin diffusion or chemical exchange. These docking simulations were therefore able to determine the general nature of the oligomerization interface whose resolution is necessarily limited by the small number of restraints and by the absence of structural information for the N-terminal region. One cluster of docking solutions using full length VirB7_XAC2622_ is shown in Figure 2D.

To investigate whether VirB7_XAC2622_24–139_ forms dimers or higher-order oligomers, glutaraldehyde cross-linking experiments were performed. These assays showed that VirB7_XAC2622_24–139_ forms dimers, trimers and higher order oligomers (Figure S4A). When the cross-linking experiment was performed in the presence of 1% SDS, no covalently cross-linked oligomers were observed, indicating that oligomerization requires the presence of a correctly folded protein (Figure S4A). As expected, no cross-links were observed when the experiment was performed using VirB7_XAC2622_51–134_ which lacks the disordered N-terminal region (Figure S4B).

### VirB7_XAC2622_ Interaction with VirB9_XAC2620_

To analyze this interaction, VirB7_XAC2622_24–139_His_ was titrated into a solution of VirB9_XAC2620_34–255_ (full-length VirB9_XAC2620_ minus the first 33 residues including a predicted signal peptide) and changes in intrinsic fluorescence were monitored. A monotonic increase in fluorescence emission was detected until saturation at a 1∶1 VirB7∶VirB9 stoichiometry (Figure S5). A dissociation constant of 4×10^−8^ M for the complex was estimated by fitting the observed fluorescence changes to a binding isotherm (equation 2, Materials and Methods).

To study this interaction by NMR, the ^15^N-HSQC spectrum of ^15^N-VirB7_XAC2622_24–139_ was acquired in the presence of unlabeled VirB9_XAC2620___34–255_. At a 1∶1 molar ratio, essentially no cross peaks were detected, except for those derived from the flexible N- and C-terminal residues (amino acids 24–25 and 134–139; data not shown). This observation is consistent with the formation of a tight complex with long correlation time, with the majority of cross peaks beyond detection.

**Figure 3:**
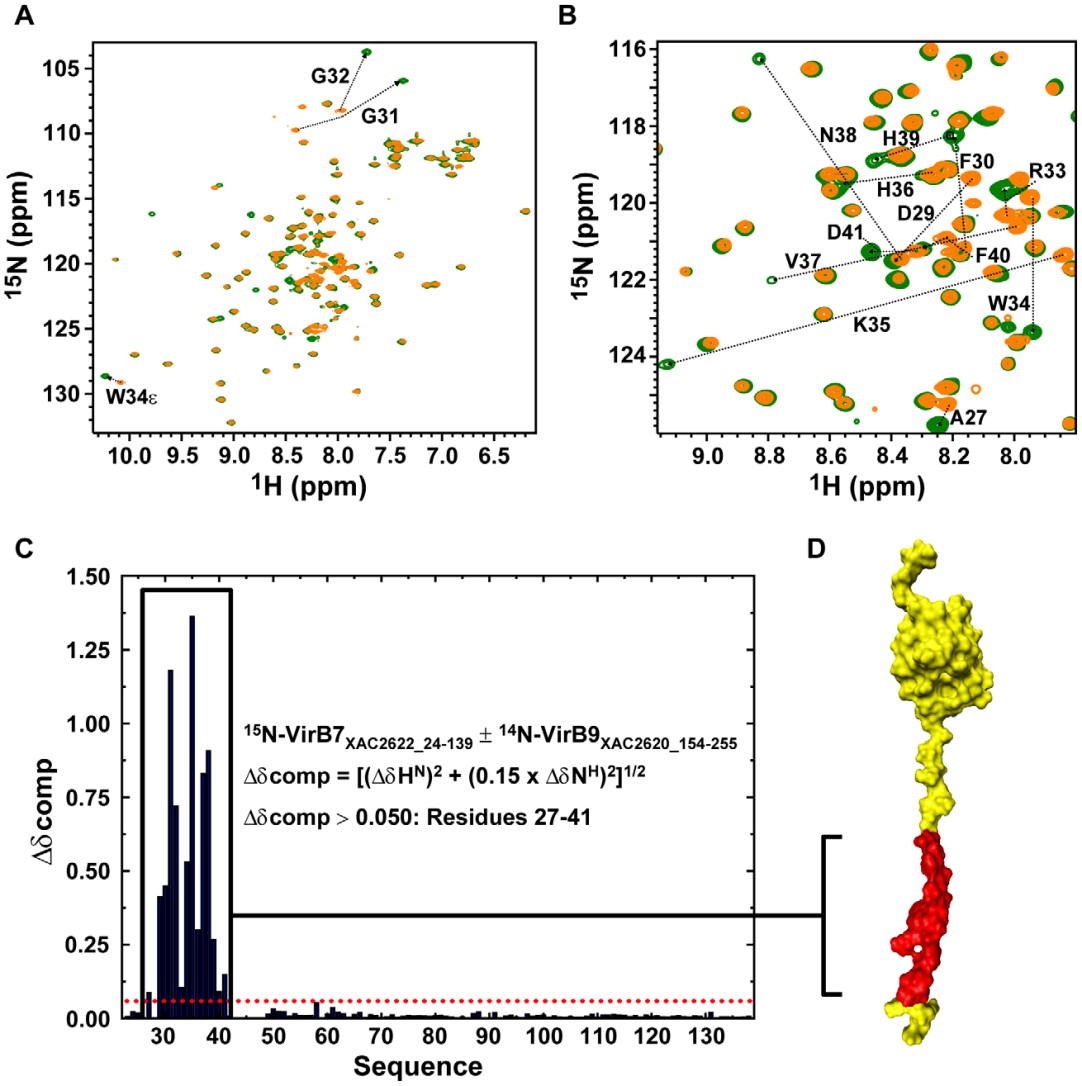
The VirB7_XAC2622_ N-terminus recognizes the VirB9_XAC2620_ C-terminal domain. (A) Superposition of the ^15^N-HSQC spectra of ^15^N-VirB7_XAC2622_24–139_ alone (orange) and in the presence of ^14^N-VirB9_XAC2620_154–255_ (green). (B) Same as A, but with a close-up view of the central spectral region. The residues for which significant changes in peak positions were observed upon complex formation are indicated. (C) Weighted chemical shift changes (Δδcomp) of VirB7_XAC2622_ upon binding to VirB9_XAC2620_. (D) Residues affected by interaction with VirB9_XAC2620_154–255_ (residues 27–41; red) are color coded on the structure of VirB7_XAC2622_24–139_.

VirB7_XAC2622_ was previously shown to interact with the C-terminal domain of VirB9_XAC2620_ [15] as has been shown for other VirB7-VirB9 homologs [32], [35]. We therefore produced a fragment, VirB9_XAC2620_154–255_, corresponding to the C-terminal domain of VirB9_XAC2620_. Changes in the ^15^N-HSQC spectrum of VirB7_XAC2622_24–139_ upon adding VirB9_XAC2620_154–255_ showed that the binding occurs in slow exchange on the NMR time scale, consistent with the sub-micromolar affinity detected by fluorescence spectroscopy for the interaction with full length VirB9 (VirB9_XAC2620_34–255_). In order to map the VirB9 binding site on the structure of VirB7, we assigned the backbone resonances of VirB7_XAC2622_24–139_ in complex with VirB9_XAC2620_154–255_ and analyzed the chemical shift differences (Figure 3A and B). This analysis showed that only residues 27–41, within the disordered VirB7 N-terminus, undergo significant chemical shift perturbations (Figure 3C and D). This region is adjacent to, but does not overlap, the N-terminal region involved in VirB7_XAC2622_ oligomerization (residues 42–49). ^1^H-^15^N RDCs and backbone chemical shifts for VirB7_XAC2622_24–139_ alone and in the presence of VirB9_XAC2620_154–255_ are essentially identical, with the exception of the N-terminal region that is involved in binding to VirB9 (Figure 3 and Figure S6). Thus, the folded domain does not participate in the recognition of the VirB9_XAC2620_ C-terminal domain and the linker between the VirB9 interaction site and the VirB7 globular domain is flexible enough to permit the two regions to align independently in the alignment medium. Interestingly, VirB7_XAC2622_24–139_ is able to simultaneously oligomerize and interact with VirB9_XAC2620_154–255_, as the cross-peaks related to the regions involved in VirB7 oligomerization shift even in the presence of VirB9_XAC2620_154–255_ (Figure S7).

### VirB7_XAC2622_-Induced Changes in the VirB9_XAC2620_ C-terminal Domain

**Figure 4:**
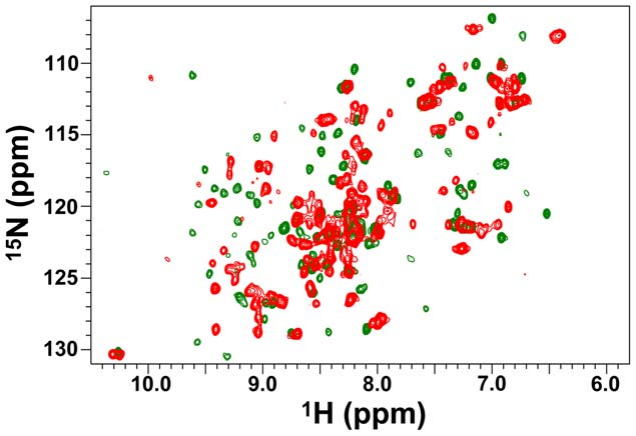
The conformation of the VirB9_XAC2620_ C-terminal domain changes significantly upon interacting with VirB7_XAC2622_. ^15^N-HSQC spectra of ^15^N-VirB9_XAC2620_154–255_ in the absence (red) and in the presence (green) of ^14^N-VirB7_XAC2622_24–139_. Spectra were collected at 30°C on a 500 MHz spectrometer equipped with a room temperature probe.

The ^15^N-HSQC spectrum of VirB9_XAC2620___154–255_ showed characteristics of poor line shape and chemical shift dispersion (Figure 4; red) suggestive of conformational disorder and a probable lack of stable tertiary structure. However, when unlabeled VirB7_XAC2622_24–139_ was added to the solution containing ^15^N-VirB9_XAC2620_154–255_, the ^1^H-^15^N cross peaks became more uniform in shape and more highly resolved (Figure 4; green). These observations suggest that VirB9_XAC2620_154–255_ on its own is in a dynamic conformational equilibrium or not properly folded in the absence of VirB7_XAC2622_24–139_. Nevertheless, it undergoes a significant conformational change which greatly reduces its conformational flexibility when bound to the latter protein.

In order to further investigate whether the interaction of VirB9_XAC2620_154–255_ with the N-terminal domain of VirB7_XAC2622_ alone is sufficient to drive the formation of a specific VirB7-VirB9 complex, we studied the interaction of ^15^N-labeled VirB9 with an unlabeled peptide derived from the VirB7_XAC2622_ N-terminus (residues 24–46), which includes the VirB7_XAC2622_ N-terminal segment that interacts with VirB9_XAC2620_ (amino acids 27–41). Indeed, the perturbations observed in the ^15^N-HSQC spectra of VirB9_XAC2620_154–255_ in the presence of the full-length VirB7_XAC2622_24–139_ or the VirB7_XAC2622_24–46_ peptide are essentially the same (Figure 4 and Figure S8), demonstrating that the VirB7 N-terminal region is sufficient to interact with the VirB9_XAC2620_ C-terminal domain.

To investigate whether aggregation could be responsible for the line broadening observed in the ^15^N-HSQC spectrum of VirB9 C-terminal domain, we calculated the approximate overall rotational correlation time (τ_c_) from estimates of ^15^N T_1_ and T_2_ obtained using 1D versions of the ^15^N relaxation experiments. The values of τ_c_ of VirB9_XAC2610_154–255_ alone or in complex with the VirB7_XAC2622_24–46_ peptide are approximately 6.5 ns (data not shown), which suggest that the VirB9_XAC2620_ C-terminal domain is monomeric in both conditions.

### Relaxation Studies of VirB7_XAC2622_24–139_

**Figure 5:**
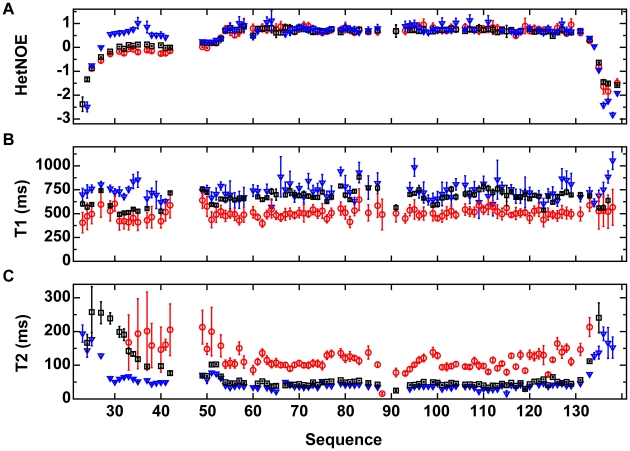
^15^N relaxation data for VirB7_XAC2622_24–139_. (A) Heteronuclear {^1^H}-^15^N NOE (HetNOE), (B) ^15^N-T1 and (C) ^15^N-T2 relaxation rates as a function of the protein sequence. Black squares: ^15^N-VirB7_XAC2622_24–139_ at 800 µM; red circles: ^15^N-VirB7_XAC2622_24–139_ at 100 µM; blue inverted triangles: ^15^N-VirB7_XAC2622_24–139_ – ^14^N-VirB9_XAC2620_154–255_ complex at 400 µM. Data are presented as mean ± uncertainty of the fitted parameter. Relaxation parameters for residues involved in oligomerization (43–47 and some between positions 86 and 93) were not obtained because they were not detectable in the ^15^N HSQC spectrum at high protein concentration (800 µM) and display amplitudes too low to allow precise measurements at lower concentrations.

Measurements of ^15^N relaxation times (T_1_ and T_2_) and heteronuclear {^1^H}-^15^N NOE were performed for ^15^N-VirB7_XAC2622_24–139_ alone (at 100 and 800 µM) and in complex with unlabeled VirB9_XAC2620_154–255_. The relaxation data are consistent with the presence of a flexible N-terminal tail and a more rigid globular domain (Figure 5). It is worth noting that the VirB9 binding surface of VirB7_XAC2622_24–139_ (residues 27–41) becomes less flexible upon binding to VirB9 (Figure 5A and C). In the absence of VirB9, residues 29–42 display {^1^H}-^15^N heteronuclear NOE values close to zero, indicating that this segment is not fully flexible and may present transient structures poised to interact with VirB9. Changes in ^15^N-T_1_ and ^15^N-T_2_ observed upon raising the VirB7_XAC2622_24–139_ concentration from 100 to 800 µM indicate a significant decrease in the overall rotational correlation rate, consistent with the formation of oligomers (Figure 5B and C). Relaxation parameters for residues involved in oligomerization (43–47 and some between positions 86 and 93) were not obtained because they were not detectable in the ^15^N-HSQC spectrum at high protein concentration (800 µM). Although these cross peaks become visible upon diluting the sample, they display too low amplitude to allow precise measurements. From the ^15^N T_1_ and T_2_ data, the overall rotational correlation times of VirB7_XAC2622_ at the concentrations of 100 and 800 µM were estimated to be 5.8 and 12.6 ns, respectively. These values are consistent with the predominance of a monomer at lower concentration and a dimer at higher concentration. This observation, however, does not exclude the possibility of the existence of an equilibrium which includes higher-order oligomers, as shown by glutaraldehyde cross-linking data.

### VirB7_XAC2622_ Contributes to VirB9_XAC2620_ and VirB10_XAC2619_ Stability *in vivo*

**Figure 6:**
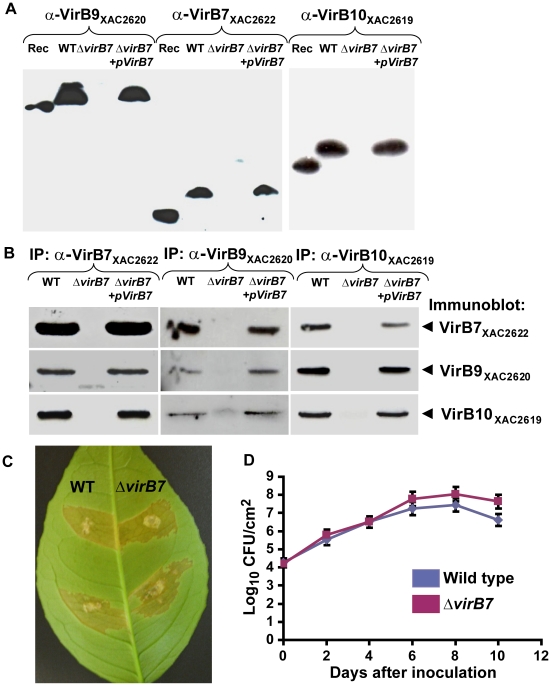
A complex between VirB7_XAC2622_, VirB9_XAC2620_ and VirB10_XAC2619_ is formed *in vivo* in Xac cells. (A) Immunoblot analysis of VirB9_XAC2620_, VirB7_XAC2622_ and VirB10_XAC2619_ protein levels in bacterial total protein extracts. Polyclonal antibodies raised against VirB9_XAC2620_ (α-VirB9_XAC2620_), VirB7_XAC2622_ (α-VirB7_XAC2622_) and VirB10_XAC2619_ (α-VirB10_XAC2619_) were used. Rec: purified recombinant proteins used as positive controls; WT: Wild type Xac strain; Δ*virB7*: Δ*virB7_XAC2622_* knockout strain and Δ*virB7*+*pVirB7*: Δ*virB7_XAC2622_* knockout cells complemented with the pUFR-VirB7 plasmid. Recombinant VirB7_XAC2622_24–139_ has a slightly greater electrophoretic mobility than native VirB7_XAC2622_, possibly due to the presence of a covalently attached lipid moiety or retention of the signal sequence of the native protein. VirB9_XAC2620_34–255_ (26 kDa) and VirB10_XAC2619_85–389_His_ (34 kDa) are also smaller than the respective native proteins. (B) Coimmunoprecipitation assays detecting reciprocal interactions between VirB proteins. Xac total cell lysates were immunoprecipitated (IP) with anti-VirB7_XAC2622_, anti-VirB9_XAC2620_ or anti-VirB10_XAC2619_ serum and the presence of VirB7, VirB9 or VirB10 was detected by immunoblot analysis. Lane labels are the same as in part (A). No VirB proteins were detected in control experiments using pre-immune sera (data not shown). (C) Characterization of the Δ*virB7_XAC2622_* gene knockout in *Citrus sinensis* infections. Macroscopic symptoms on abaxial surface of orange leaves 12 days post inoculation with wild type (left) and Δ*virB7* (right) strains of Xac. (D) Growth curves of Xac strains on orange leaves. Blue: wild type; red: Δ*virB7*. Data are presented as mean ± standard deviation.

In *Agrobacterium tumefaciens* cells, the VirB7 protein contributes to VirB9 and VirB10 stability [42]. We therefore produced a *xac2622* gene knockout strain and performed immunoblot experiments to assess the expression of VirB7_XAC2622_, VirB9_XAC2620_ and VirB10_XAC2619_. The production of all three proteins could be detected in wild type cells but none could be detected in the Δ*virB7_XAC2622_* strain (Figure 6A). To evaluate if the absence of VirB9_XAC2620_ and VirB10_XAC2619_ in the mutant strain is due to the lack of the VirB7_XAC2622_ production or to a polar effect in the T4SS operon, Δ*virB7_XAC2622_* cells were complemented with a plasmid encoding the VirB7_XAC2622_ protein in trans. The expression of VirB7_XAC2622_ from the pUFR-VirB7 plasmid restored VirB9_XAC2620_ and VirB10_XAC2619_ protein levels (Figure 6A). Furthermore, no significant differences in *virB9_XAC2620_* transcript levels were detected between the wild-type, Δ*virB7_XAC2622_* and Δ*virB7_XAC2622_*+pUFR-VirB7 strains in quantitative RT-PCR experiments (data not shown). These results show that VirB7_XAC2622_ is necessary for the stability of VirB9_XAC2620_ and VirB10_XAC2619_ proteins *in vivo*.

The VirB7, VirB9 and VirB10 orthologs TraN, TraO and TraF form a stable trimeric complex [33], [34]. In order to determine whether the VirB7_XAC2622_, VirB9_XAC2620_ and VirB10_XAC2619_ form a stable complex in Xac cells, we performed immunoprecipitation experiments using antisera for each of the three proteins. Results described in Figure 6B show that VirB7_XAC2622_, VirB9_XAC2620_ and VirB10_XAC2619_ are present in the material immunoprecipitated by each of the three antisera in wild-type cells. Furthermore, none of the three proteins were immunoprecipitated from Δ*virB7_XAC2622_* cells while all three proteins were detected in immunoprecipitation experiments using Δ*virB7_XAC2622_*+pUFR-VirB7 cells (Figure 6B). Altogether, these results show that a complex between VirB7_XAC2622_, VirB9_XAC2620_ and VirB10_XAC2619_ is formed *in vivo* in Xac cells.

In order to test the role of VirB7_XAC2622_ in Xac virulence, sweet orange leaves were infiltrated with the wild type and Δ*virB7_XAC2622_* strains. Both strains presented essentially the same infection phenotypes, including water-soaking, hyperplasia and necrosis (Figure 6C) [13]. *In planta* growth curves of the wild type and Δ*virB7_XAC2622_* cells revealed that the two strains replicate at a similar rate (Figure 6D). These data indicate that the VirB7_XAC2622_ protein does not participate in the Xac's ability to induce canker symptoms in orange leaves under the experimental conditions tested. Similarly, a T4SS knockout failed to affect the pathogenicity of *X. campestris* pv. campestris on several host plants [43]. Xac pathogenicity in citrus and canker symptoms are strongly dependent on the action of virulence factors secreted by the Type III secretion system [13]. The T4SS is probably involved in other, as yet unidentified, cellular functions. As noted in the Introduction, the Xac pXAC64 megaplasmid codes for a second T4SS probably involved in plasmid mobilization [15], [27] and whose structural components exhibit only very low sequence identity to their counterparts in the chromosomally encoded T4SS under study. For example, its VirB9 and VirB10 homologs (XACb0039 and XACb0038) are only 23–24% identical to VirB9_XAC2620_ and VirB10_XAC2619_ [15]. Furthermore, the pXAC64 plasmid does not code for any proteins with similarity to VirB7_XAC2622_ or any other known VirB7 proteins [15]. Therefore, it is unlikely that the two Xac T4SSs exercise redundant functions.

## Discussion

The structure of the outer membrane complex of the T4SS coded by the conjugative *E. coli* plasmid pKM101 has been determined by X-ray crystallography and consists of 14 repeats of the TraN – TraO_CT_ – TraF_CT_ trimer [34], homologs of VirB7, the C-terminal domain of VirB9 and the C-terminal domain of VirB10, respectively. This structure revealed that the inner lining of the channel is formed by the C-terminal domains of the 14 TraF subunits. The TraN-TraO subcomplexes surround the internal ring formed by TraF. The 33 residue long lipidated TraN subunit is the outermost subunit of the complex whose external diameter is 172 Å [34].

### *Xanthomonas* VirB7 Structure and Interactions in the Context of the T4SS Core Complex

**Figure 7:**
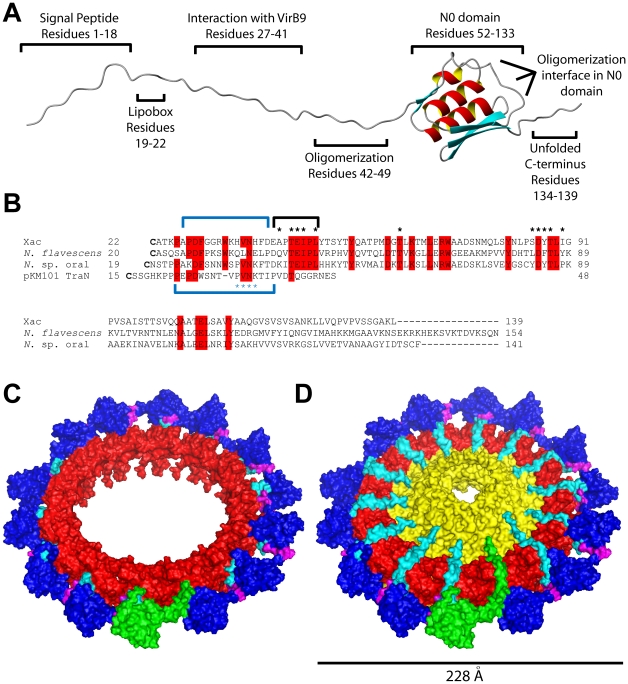
VirB7_XAC2622_ structure and interactions in the context of the T4SS outer membrane complex. (A) Schematic organization of the VirB7_XAC2622_ protein, based on bioinformatics and experimental data. (B) Sequence alignment of the VirB7 proteins from *Xanthomonas citri* subsp. citri (GenBank code AAM37471), *Neisseria flavescens* (EER57212), *Neisseria* sp. oral taxon 014 str. F0314 (EFI23373) and TraN from pKM101 (AAA86456). Sequences begin at the cysteine residues of the predicted lipidation sites. Black asterisks indicate residues involved in observed intermolecular NOEs. Blue asterisks indicate the PVNK motif found in the pKM101 TraN protein. Blue brackets above and below the alignment indicate the VirB9_XAC2620_ and TraO binding sites in VirB7_XAC2622_ and TraN, respectively. The black bracket indicates the N-terminal oligomerization site. Residues outlined in red are conserved in at least three of the four sequences. Note that the codon for V55 of the protein from *Neisseria flavescens* is incorrectly assigned as a start codon in the GenBank database. (C) Final molecular dynamics configuration of the (VirB7_XAC2622_37–139_-TraO_CT_)_14_ complex. TraO_CT_ is represented in red, VirB7_XAC2622_ is in cyan (residues 37–41), magenta (residues 42–49) and dark blue (residues 50–139). One VirB7_XAC2622_37–139_ unit is shown in green. (D) Putative model for the VirB7_XAC2622_-VirB9_XAC2620_-VirB10_XAC2619_ outer membrane complex produced by adding TraN residues 19–32 (representing VirB7_XAC2622_ residues 22–36, see alignment in part B) and TraF_CT_ (representing VirB10_XAC2619_) to the structure shown in part C. The color scheme is the same as in part C except that cyan represents VirB7_XAC2622_ residues 22–41 and TraF/VirB10 is shown in yellow. One complete VirB7_XAC2622_ molecule is shown in green.

VirB7_XAC2622_ is structurally distinct from all VirB7 homologs characterized to date. Its structural motifs are illustrated in Figure 7A. Like all other VirB7 proteins, it has a signal peptide, a conserved cysteine residue (Cys22) within a lipobox for covalent attachment to outer membrane lipids and a short extended region involved in interaction with VirB9. These motifs are all contained within the first 41 residues, which corresponds relatively well with the size of the majority of unprocessed VirB7 proteins (45–65 amino acids) [36].

VirB7 proteins are very poorly conserved though it has been observed that many proteins of this family have in common a PVNK motif that is involved in the interaction with VirB9 [35]. The VirB9 binding site in VirB7_XAC2622_ includes a HVNH sequence (residues 36–39) found in almost all Xanthomonadaceae orthologs (PVNR in *X. albilineans* and *Stenotrophomonas maltophilia*) which aligns with a perfect PVNK motif in the *Neisseria* sp. oral taxon 014 str. F0314 ortholog (Figure 7B). The side chains of the two most conserved residues of this motif, Val and Asn, make intimate contacts with TraO/VirB9 in the pKM101 outer membrane complex structure [35]: the Val side chain is inserted between the two TraO β-sheets while the Asn amide Oδ1 and Nδ2 atoms makes a set of three H-bonds with the TraO main chain. Furthermore, the PVNK motif in TraN is preceded by an approximately 10 amino acid segment that makes contacts with TraO, including a 3 residue β-strand that adds to the edge of one of the TraO β-sheets [34]. The corresponding regions in VirB7_XAC2622_ are precisely those that demonstrate the greatest chemical shift perturbations upon binding to VirB9_XAC2620_ (Figure 3). This suggests that interactions between VirB7_XAC2622_ and VirB9_XAC2620_ are very similar to that observed in the TraN-TraO complex.

The VirB7-VirB9 interactions described are most likely responsible for the loss of conformational flexibility observed for the N-terminal tail of VirB7_XAC2622_ and the C-terminal domain of VirB9_XAC2620_. Furthermore, interaction with VirB7_XAC2622_ is likely to be accompanied by a significant change in the structure of VirB9_XAC2620_154–255_, as revealed by the very large chemical shift perturbations observed for the majority of the ^1^H-^15^N cross-peaks (Figure 4). These changes in VirB9_XAC2620_ structure and dynamics may be related to its instability *in vivo* in the absence of VirB7_XAC2622_. Our immunoblot and coimmunoprecipitation data suggest that VirB7_XAC2622_, VirB9_XAC2620_ and VirB10_XAC2619_ form a stable trimeric complex *in vivo* and that the stabilities of VirB9_XAC2620_ and VirB10_XAC2619_ are dependent on VirB7_XAC2622_ (Figure 6). This may be a physiologically relevant mechanism by which excess VirB9 and VirB10 are degraded to maintain proper VirB7-VirB9-VirB10 stoichiometry.

VirB7_XAC2622_ has a mosaic structure (Figure 7A). In addition to the canonical VirB7-like N-terminal region, VirB7_XAC2622_ has a C-terminal region that includes a globular domain (residues 52–133). This globular domain does not interact with VirB9_XAC2620_154–255_ but does interact with an extended region (residues 42–49) that immediately precedes the globular domain in another VirB7_XAC2622_ molecule (Figure 2D), leading to the formation of homo-oligomers. The unique structural features of VirB7_XAC2622_ raise the question of how they may be incorporated into the model for the T4SS outer membrane complex tetradecamer previously solved for the pKM101 conjugation machine [34]. Specifically: can VirB7_XAC2622_ oligomerization occur while maintaining VirB7-VirB9 interactions in the context of the pore? And how are the VirB7_XAC2622_ globular domains oriented with respect to the pore? In order to investigate these questions, we used the VirB7_XAC2622_-TraN alignment depicted in Figure 7B to manually place 14 different NMR models of residues 37–139 of VirB7_XAC2622_ (VirB7_XAC2622_37–139_) around a tetradecameric ring made up of TraO_CT_ subunits derived from the pKM101 TraN-TraO_CT_-TraF_CT_ structure [34]. The VirB7_XAC2622_37–139_ subunits were placed so that Val37 and Asn38 occupied positions similar to Val33 and Asn34 of TraN. This (VirB7_XAC2622_37–139_-TraO_CT_)_14_ complex was used as a starting model in a molecular dynamics simulation in which the following restraints were used: i) The 14 TraO_CT_ subunits were fixed in space, ii) VirB7_XAC2622_ residues 37 and 38 were fixed in space and iii) intermolecular NOE derived distance restraints were placed on 5 hydrogen pairs between neighboring VirB7_XAC2622_ subunits. During the initial cycles of the molecular dynamics simulation, the distance observed between the 13 intermolecular VirB7_XAC2622_ hydrogen pairs for which NOEs were measured (Figure 2D and Results above) decreases from the 12–25 Å range observed in the starting model to a 4–7 Å range, consistent with observation of NOE signals. These short contact distances are then observed throughout the simulation for all 14 VirB7_XAC2622_ pair interfaces made up of residues A43, T45, E46, I47, L49 in one VirB7_XAC2622_ molecule and residues T63, S85, D86, Y87, T88, I90 in a neighboring VirB7_XAC2622_. This is a consequence of the strong distance restraints included in the potential energy function. The final molecular dynamics configuration is shown in Figure 7C. Adding models of TraN residues 19–32 to represent VirB7_XAC2622_ residues 22–36 (see alignment in Figure 7B) and TraF_CT_ (to represent VirB10_XAC2619_) results in the structure shown in Figure 7D made up of 14 repeats of VirB7_XAC2622_37–139_, TraN_19–32_, TraO_CT_ and TraF_CT_. This can be seen as putative model for the VirB7_XAC2622_-VirB9_XAC2620_-VirB10_XAC2619_ outer membrane complex. Note that in Figures 7C and 7D, the VirB7 N0 domains (dark blue) adopt a variety of orientations with respect to the central ring and the membrane normal. This flexibility is derived from the conformational freedom of the VirB7_XAC2622_ regions immediately before and after residues 42–49 (magenta) involved in VirB7-VirB7 interactions.

Our proposal of a physiological role for VirB7_XAC2622_ oligomerization is supported by an analysis of conserved residues in the small group of full-length VirB7_XAC2622_ homologs found in Xanthomonadaceae and the two *Neisseria* species *Neisseria flavescens* SK114 and *Neisseria* sp. oral taxon 014 str. F0314. An alignment of VirB7_XAC2622_ and the two *Neisseria* proteins is shown in Figure 7B. The three proteins have only 21% sequence identity. However, 6 out of 11 of the residues involved in intermolecular NOEs at the B7-B7 interface (E46, I47, L49, T63, D86 and T88) are absolutely conserved in all Xanthomonadaceae, *Neisseria* str. F0314 and *Neisseria flavescens* and is either Phe or Tyr at position 87. Thus, the oligomerization interface is well conserved in an otherwise highly diverse protein family.

VirB7_XAC2622_ oligomerization is different from the dimerization observed in the canonical VirB7 protein from *A. tumefaciens* [30] which occurs via the formation of disulfide bonds involving residue 24. The structure of the outer membrane complex [34] however, is not compatible with the maintenance of these disulfide bonds in a fully assembled T4SS. Furthermore, VirB7_XAC2622_ does not have any cysteine residues other than at the N-terminal lipidation site.

### The VirB7_XAC2622_ Globular Domain is Structurally Similar to Proteins Associated with Transport Across Bacterial Outer Membranes

**Figure 8:**
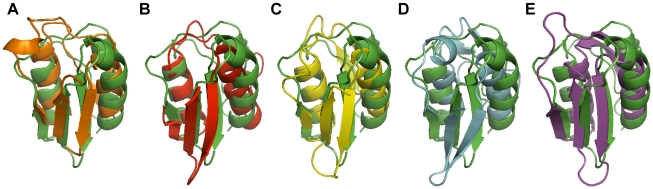
Superposition of the structure of the VirB7_XAC2622_ N0 domain with structural homologs. The VirB7_XAC2622_ globular domain (residues 51–134; green) is superposed with (A) the signaling domain of PupA (orange, residues 3–76, PDB: 2A02); (B) the N0 domain of GspD (red, amino acids 3–79, PDB: 3EZJ); (C) the N0 domain of EscC (yellow, amino acids 31–103, PDB: 3GR5), (D) residues 97–176 of gene product 44 from bacteriophage Mu (light blue, PDB: 1WRU) and (E) residues 81–161 of DotD from *L. pneumophila* (purple, PDB: 3ADY).

The VirB7_XAC2622_ globular domain has no significant sequence similarity to proteins of known structure. We therefore used the Dali Server [44] to search for proteins with similar topology to this domain. This analysis revealed that the VirB7_XAC2622_ folded region resembles domains found in the following proteins (Table 3 and Figure 8): i) the TonB-dependent receptors (periplasmic signaling domain; Figure 8A) [45], [46], ii) the outer membrane secretin channel GspD from the type II secretion system (T2SS; Figure 8B) [47], iii) the secretin EscC from the Type III secretion system (T3SS; Figure 8C) [48], iv) the needle-like cell-puncturing device components gp27 and gp44 from long-tailed phages like T4 and Mu (Figure 8D) [49], [50] and v) the Type VI secretion system (T6SS) protein VgrG (superposition not shown) [51]. These domains are found at the N-termini of T2SS and T3SS secretins and have been denominated N0 domains which have also been identified in the Type IV pilus secretin PilQ [47] and the filamentous phage secretin pIV [52]. It is striking that in spite of its small and compact nature, domains with the VirB7_XAC2622_ topology are found only in a restricted number of proteins, all of which are involved in the transport of molecules across bacterial outer membranes. These observations suggest that all of these proteins may be distantly related and have evolved in the periplasm or outer membrane to adopt a variety of functions, from structural modules in outer membrane pores (secretins from type II and type III secretion systems, type IV pili and filamentous phages) to membrane-penetrating devices in T6SS and long-tailed bacteriophages, and signal-transduction modules in TonB-dependent receptors.

**Table 3:** Structural similarity of the VirB7_XAC2622_ globular domain^a^.

PDB	Description	XR^b^ - Z-score/RMSD (Å)	NMR^c^ - Z-score/RMSD (Å)
2A02	PupA – TBDRSD	7.0/2.5	7.3/3.0
1ZZV	FecA – TBDRSD	6.5/2.9	6.8/2.6
3GR5	EscC – T3SS	6.2/2.9	6.1/3.0
3ADY	DotD – T4SS-B	6.1/3.1	5.7/2.9
1WRU	gp44 – Mu phage	6.1/3.3	5.7/3.9
2IAH	FpvA – TBDRSD	6.0/2.5	6.0/2.7
3EZJ	GspD – T2SS	5.2/2.7	5.0/2.8

^*a*^Structural comparisons were done using the DALI-server [44].

^*b*^XR: Analysis performed with the X-ray crystal structure of VirB7_XAC2622_51–134_.

^*c*^NMR: Analysis performed with the lowest energy NMR model of VirB7_XAC2622_24–139_ (residues 51–134). TBDRSD: TonB-dependent receptor signaling domain.

Nakano et al. [53] have recently described the crystal structure of the DotD lipoprotein of the Type IV-B secretion system from *Legionella pneumophila*. Type IV-B secretion systems have weak or no sequence similarity with the larger group of Type IV-A secretion systems [54] to which the Xanthomonad T4SS pertains. Interestingly, the DotD crystal structure presents a C-terminal region with an N0 domain topology similar to that of VirB7_XAC2622_ structure (Figure 8E), though the proteins present no significant sequence similarity. An interesting difference between the two structures is that a 6 amino acid sequence (called the “lid”) of the otherwise disordered N-terminal region is visible in the crystal structure and makes β-strand addition to β1. This interaction is different from that observed in the VirB7_XAC2622_ oligomer described here. Since no electron density for the 23 residues between the lid and the N0 domain were visible, it was not clear whether they come from the same molecule in the crystal lattice. We therefore predict that DotD will eventually be shown to exhibit the same characteristics as VirB7_XAC2622_, namely to interact with both a VirB9-like protein and to form head-to-tail polymeric rings based on interactions between the flexible N-terminal region and globular N0 domains of neighboring subunits. This analysis points to previously unrecognized common structural features between the outer membrane complexes of Type IV-A and Type IV-B secretion systems.

The novel VirB7_XAC2622_ structure, its oligomerization and its interaction with VirB9 described here point to a possible structural variation in the *Xanthomonas* T4SS that could result in the formation of an extra ring layer in the core complex. While the VirB7-VirB7 interactions observed in the NMR experiments were of relatively low affinity (fast exchange chemical shift changes were observed in the 7–850 µM range), these interactions would be strengthened by the increased effective concentration afforded by the fourteen neighboring VirB7_XAC2622_ binding sites on the outer surface of the VirB9/VirB10 ring. Several hypotheses regarding the physiological role of this VirB7_XAC2622_ ring can be proposed. One possibility is that the weak B7-B7 interactions break and reform again to accommodate different conformational states of the outer pore during substrate translocation or to permit allosteric communication between the outer and inner membrane core complexes. Another possibility is that the VirB7_XAC2622_ N0 domains could act as a conduit for signal transmission between substrates or signaling molecules in the periplasm and the VirB9 and VirB10 subunits. Whether the VirB7_XAC2622_ N0 domain carries out a purely structural function or is involved in substrate recognition or signal transduction will have to be tested in the future.

## Materials and Methods

### Cloning, Protein Expression and Purification

Plasmids, oligonucleotides and bacterial strains used in this study are listed in Table S1. The DNA fragments encoding residues 24–139 of VirB7_XAC2622_ (GenBank AAM37471), 154–255 of VirB9_XAC2620_ (GenBank AAM37469) and 85–389 of VirB10_XAC2619_ (GenBank AAM37468) were amplified by PCR from *Xanthomonas citri* subsp. citri strain 306 genomic DNA and inserted into the pET28a expression vector (Novagen) using the *Nde*I and *Xho*I (VirB7_XAC2622_) or *Nde*I and *Bam*HI (VirB9_XAC2620_ and VirB10_XAC2619_) cloning sites, to express the recombinant proteins fused with an N-terminal His-tag. DNA fragments encoding residues 51–134 of VirB7_XAC2622_ and 34–255 of VirB9_XAC2620_ were cloned into the pET11a vector (Novagen) using *Nde*I and *Bam*HI sites. All constructs were confirmed by DNA sequencing. *E. coli* BL21(DE3)RP and BL21(DE3)Star strains (Novagen) were transformed with the recombinant plasmids for expression of the protein products which are referred to as VirB7_XAC2622_24–139_His_, VirB9_XAC2620_154–255_His_, VirB7_XAC2622_51–134_, VirB9_XAC2620_34–255_ and VirB10_XAC2619_85–389_His_. Cells were grown in 2XTY for expression of unlabeled proteins or in M9 minimal media containing ^15^NH_4_Cl and ^12^C-glucose or ^13^C-glucose (Cambridge Isotope Laboratories) for production of ^15^N or ^15^N and ^13^C isotopically labeled proteins. Recombinant protein expression was induced at midlog phase by the addition of 0.5 mM isopropyl *β*-D-1-thiogalactopyranoside and the cells were grown for four hours at 37°C. Cells were then harvested and lysed by French Press. In the case of VirB7_XAC2622_24–139_His_ and VirB9_XAC2620_154–255_His_, the lysis supernatants were separated by passage through Q-Sepharose (VirB7_XAC2622_24–139_His_) or SP-Sepharose (VirB9_XAC2620_154–255_His_) ion exchange columns (GE Healthcare) followed by affinity chromatography using a nickel affinity column. The N-terminal His-tags were removed using the Thrombin CleanCleave kit (Sigma), creating the proteins VirB7_XAC2622_24–139_ and VirB9_XAC2620_154–255_. A final purification step consisted of passage through a Superdex 75 26/60 size exclusion column (GE Healthcare). VirB7_XAC2622_51–134_ was purified using Q-sepharose anion exchange and Superdex 75 gel filtration chromatography. VirB9_XAC2620_34–255_ and VirB10_XAC2619_85–389_His_ are expressed as insoluble proteins present in inclusion bodies. After lysis, VirB9_XAC2620_34–255_ was solubilized in the presence of 8 M urea and purified by SP-Sepharose cation exchange and Superdex 75 size exclusion chromatographies using buffers containing 8 M urea. VirB10_XAC2619_85–389_His_ was purified by affinity chromatography using buffers containing 8 M urea. Purified proteins were refolded by successive dialysis against decreasing concentrations of urea in 10 mM sodium acetate (pH 5.0) for VirB9_XAC2620_34–255_ or 10 mM Tris-HCl (pH 8.0), 150 mM NaCl for VirB10_XAC2619_85–389_His_. Synthetic peptides corresponding to VirB7_XAC2622_24–46_ and VirB7_XAC2622_38–52_ were purchased from Biomatik Corporation (Cambridge, Ontario, Canada) and EZBiolab (Carmel, Indiana, USA), respectively.

### NMR Spectroscopy

NMR samples containing ^15^N or ^15^N/^13^C labeled protein were prepared in 10 mM ^2^H-sodium acetate (pH 5.0), containing 50 mM sodium chloride, 0.05% (w/v) sodium azide, 7% (v/v) ^2^H_2_O and 1 mM trimethylsilyl-2,2,3,3-tetradeuteropropionic acid as internal standard for ^1^H chemical shift referencing. NMR spectra were acquired on a 600 MHz Varian Unity-Inova spectrometer equipped with inverse triple resonance (^1^H, ^15^N, ^13^C) cold probe, or on BRUKER 500 MHz DRX or 800 MHz Avance III spectrometers equipped with inverse triple resonance (^1^H, ^15^N, ^13^C) room temperature probes. Unless otherwise mentioned, all NMR experiments were performed at 40°C. The backbone resonance assignment of ^15^N/^13^C-VirB7_XAC2622_24–139_ was achieved by analyzing the following three-dimensional spectra: HNCA, HN(CO)CA, HNCO, HN(CA)CO, HNCACB and CBCA(CO)NH [55]. Side-chain resonance assignments were determined by analyzing the following spectra: 2D ^13^C-HSQC, 2D TOCSY, 2D NOESY, 3D HNHA, 3D HBHA(CBCACO)NH, 3D ^15^N-TOCSY-HSQC, 3D H(CCO)NH-TOCSY, 3D (H)CC(CO)NH-TOCSY, 3D ^15^N-NOESY-HSQC, 3D H(C)CH-TOCSY and 3D ^13^C-NOESY-HSQC (specific for the aliphatic or the aromatic regions). The 3D H(C)CH-TOCSY and 3D ^13^C-NOESY-HSQC spectra were recorded in >99% (v/v) ^2^H_2_O. NOESY experiments were carried out using 0.2–0.3 mM protein samples and all NOESY mixing times were 100 ms. Backbone resonance assignments for ^15^N/^13^C-VirB7_XAC2622_24–139_ in complex with unlabeled VirB9_XAC2620_154–255_ were obtained from the analysis of three-dimensional HNCA, HN(CO)CA, HNCO, HNCACB, CBCA(CO)NH, HBHA(CBCACO)NH and ^15^N-NOESY-HSQC experiments recorded on a sample of ^15^N/^13^C-VirB7_XAC2622_24–139_ in the presence of a 20% molar excess of unlabeled VirB9_XAC2620_154–255_. The backbone resonance assignment (^15^N and ^1^H) for VirB7_XAC2622_51–134_ was performed by comparison with the ^15^N-HSQC spectrum of the larger construct, VirB7_XAC2622_24–139_. Residual ^1^D_NH_ dipolar couplings for VirB7_XAC2622_24–139_ (0.25 mM), alone or in complex with VirB9_XAC2620_154–255_, were determined from the differences in ^1^H-^15^N splittings obtained in isotropic and anisotropic media. The ^1^H-^15^N splittings were extracted from a series of J-modulated 2D ^1^H-^15^N-HSQC experiments acquired at 30°C on the 500 MHz spectrometer, and analyzed essentially as described [56]. Residual alignment was induced by a liquid crystalline medium consisting of 5% penta-ethyleneglycol monododecyl ether (C12E5)/n-hexanol (*r* = 0.96) [57]. Fittings of RDCs to the NMR and X-ray structures were carried out using PALES [58] or MODULE [59]. All NMR spectra were processed with NMRPipe [60] and analyzed with CCPN Analysis [61]. Resonance assignments were deposited in the Biological Magnetic Resonance Data Bank (BMRB accession code 17257).

### NMR Structure Calculation

Structure calculation and automated NOE assignment were performed using CYANA 2.1 [37]. Chemical shifts of ^1^Hα, ^13^Cα, ^13^Cβ, ^13^C′ and ^15^N^H^ were used as input for TALOS [62] to predict ϕ and ψ dihedral angles that were subsequently used as restraints in the structure calculation. A total of 3471 NOE cross peaks were manually picked in the ^15^N-NOESY-HSQC and ^13^C-NOESY-HSQC (acquired specifically for the aliphatic or the aromatic regions) spectra and used as input for CYANA. The CYANA protocol consisted of 7 cycles of simulated annealing in torsion angle space with 10000 integration steps. In each cycle 300 conformers were calculated. The intra-residue, sequential and 10 unambiguous long range NOE cross peaks were assigned manually and imposed during all stages of the protocol. Chemical shift tolerances for automated NOE assignment were set to ±0.025 ppm in the direct ^1^H dimension, ±0.030 ppm in the indirect ^1^H dimension and ±0.25 ppm for the heteronuclei. The best 50 CYANA solutions were selected based on a target function criteria, and further refined in explicit water using CNS2.1 and HADDOCK2.0 [41]. The 1616 NOEs assigned by CYANA, the manually assigned NOEs, and the TALOS dihedral angle restraints were used as input during water refinement in HADDOCK. The best 20 lowest-energy conformers after water refinement were deposited in the Protein Data Bank (PDB code 2L4W). The quality of the final NMR structures was investigated by PROCHECK-NMR [63] and PSVS [64]. The structures were visualized using MOLMOL [65] or PYMOL (http://www.pymol.org).

### ^15^N Relaxation Measurements

Experiments for measuring backbone ^15^N longitudinal (T_1_) and transverse (T_2_) relaxation times, and the heteronuclear {^1^H}-^15^N NOE, were recorded at 40°C on a Varian Inova 600 MHz spectrometer using standard Biopack pulse sequences (Varian, Inc.). The inversion recovery delays used for sampling T_1_ relaxation were 50, 250, 450, 650, 850, 1050, 1250, 1450 and 1650 ms. The CPMG delays used for sampling T_2_ relaxation were 10, 30, 50, 70, 90, 110, 130, 150, 170, 190 and 210 ms. The ^1^H saturation period in the heteronuclear {^1^H}-^15^N NOE experiment was 3 s. All 2D spectra were acquired sequentially as matrices of 512(^1^H)×128(^15^N) complex points, and inter scan delay of 3 s. Relaxation rates were obtained by fitting the time decay of peak intensities to an exponential decay function with three fitted parameters, the relaxation rate, the intensity at time zero and an offset, using the CCPN Analysis software [61]. Overall rotational correlation times (τ_c_) were obtained either from the mean ratios of ^15^N T_1_/T_2_ relaxation rates of ^1^H-^15^N bond vectors located in regions of secondary structure using TENSOR2 [66], or from estimates of ^15^N T_1_ and T_2_ relaxation times obtained from 1D ^15^N-edited relaxation experiments, as previously described [67], [68].

### NMR Titration Experiments

Unlabeled VirB9_XAC2620_154–255_ or VirB9_XAC2620_34–255_ were titrated into a solution of ^15^N-labeled VirB7_XAC2622_24–139_ and a ^15^N-HSQC spectrum was acquired after the addition of each aliquot until a molar excess of 1.5 of unlabeled protein with respect to ^15^N-VirB7_XAC2622_24–139_. In analogous experiments ^15^N-labeled VirB9_XAC2620_154–255_ was titrated with unlabeled VirB7_XAC2622_24–139_ or VirB7_XAC2622_24–46_. VirB7_XAC2622_24–139_ oligomerization was studied by monitoring changes in the ^15^N-HSQC spectra as a function of protein concentration in the range of 7 to 850 µM. ^15^N-labeled VirB7_XAC2622_51–134_ was also titrated with unlabeled VirB7_XAC2622_38–52_. Chemical shift perturbations due to sample dilution or protein-protein interaction were obtained from the weighted chemical shift changes in the ^15^N-HSQC spectra calculated according to Eq. 1 [69]:
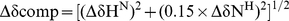
(1)where ΔδH^N^ and ΔδN^H^ are the chemical shift changes (in ppm) of the amide proton and nitrogen resonances, respectively.

### Protein-Protein Docking

Docking simulations were used to build a docking model for the dimer of VirB7_XAC2622_24–139_. The calculations were performed with HADDOCK2.0 using CNS2.1 [41], and the first model of the NMR ensemble as starting structure. The docking was driven by unambiguous intermolecular NOEs and by ambiguous interaction restraints (AIR) between active residues and active plus passive residues. The following 13 NOE restraints were used: Ala43Hβ - Ile90Hδ1; Thr45Hα - T88Hγ2; Thr45Hβ - T88Hγ2; Glu46Hβ - Tyr87Hε; Ile47Hδ1 - Thr63Hβ; Ile47Hδ1 - Thr63Hγ2; Ile47Hδ1 - T88Hα; Ile47Hδ1 - T88Hγ2; Leu49Hγ - Ser85Hα; Leu49 Hδ1 - Asp86Hα; Leu49Hδ1 - Asp86Hβ1; Leu49Hδ1 - Asp86Hβ2 and Leu49Hδ2 - Asp86Hα. Active residues were identified as those showing chemical shift perturbations of at least one standard deviation above the mean, for ^15^N-VirB7_XAC2622_24–139_ as a function of protein concentration (Figure 2). Active residues were E42, A43, T45, E46 and I47 at the N-terminal tail of the first subunit and D86, T88, I90, G91, Q115 of the C-terminal globular domain of the second subunit. Passive residues were F40, D41, P44, P48 and L49 in the N-terminal segment, and P84, S85, Y87, P92, A113 and A114 in the C-terminal domain. The N-terminal tails (residues 24–50) were kept fully flexible during all stages of the docking, while C-terminal domain residues at the interface, 84–92, 61–63 and 113–115, were maintained semi-flexible, being allowed to move only during semi-flexible refinement. The rigid body stage consisted of the calculation of 1000 solutions, from which 100 with the lowest HADDOCK scores were selected for semi-rigid simulated annealing in torsion angle space including backbone and side chain flexibility, and final refinement in Cartesian space with explicit water. The refined solutions were then clustered based on the backbone RMSD at the interface. An analogous protocol was used for docking the N-terminal (residues 24–50) and C-terminal (residues 51–139) fragments of VirB7, except that in this calculation NOE restraints were not included and flexibility was introduced only at the interface.

### Model Building and Molecular Dynamics Simulations

Models of residues 37–139 of VirB7_XAC2622_ (VirB7_XAC2622_37–139_) were positioned around a tetradecameric ring made up of TraO_CT_ subunits derived from the pKM101 T4SS TraN-TraO_CT_-TraF_CT_ structure [34]. The VirB7_XAC2622_37–139_ subunits were placed so that Val37 and Asn38 occupied positions similar to Val33 and Asn34 of TraN. Molecular mechanics optimization and dynamics were then carried out from this initial model. The OPLS/AA force-field [70] for the protein and an implicit solvent representation of the generalized Born-formalism [71] were used. In addition to the molecular force-field, distance restraints with a flat-bottom harmonic functional form were included such that an energy penalty was added to the potential when the distance between specified pairs of atoms was less than a minimum threshold value of 3 Å or exceeded a maximum of 6 Å. A harmonic force constant of 5000 kJ⋅mol^−1^⋅Å^−2^ was used. The following 5 NOE proton pairs were specified for each VirB7_XAC2622_ pair interface: Ala43Hβ - Ile90Hδ1, Thr45Hβ - T88Hγ2, Ile47Hδ1 - Thr63Hβ; Leu49Hγ - Ser85Hα; and Leu49Hδ2 - Asp86Hα. These pairs correspond to a subset of the pairs for which intermolecular NOEs were detected. Thus, a total of 70 (14×5) distance restrains were included in the potential. All ionizable residues besides histidine were treated in their charged forms. Molecular dynamics were carried out at a temperature of 300 K for a total time of 2 ns. All computations were carried out with the GROMACS 4.5.3 software [72].

### Crystallization, Data Collection and Processing

A construct corresponding to the VirB7 C-terminal region, VirB7_XAC2622_51–134_, at 14 mg/mL in 5 mM Tris-HCl pH 7.5 and 25 mM sodium chloride, was submitted to vapor diffusion sitting-drop crystallization trials at 18°C. Large plates appeared after one day over a reservoir solution comprising 1.4 M ammonium sulfate and 4% (v/v) isopropyl alcohol. Reservoir solution supplemented with 25% (v/v) glycerol was used as cryoprotectant. Crystals were flash frozen at 100 K and submitted to X-ray diffraction at beam line W01B-MX2 of the Brazilian Synchrotron Light Laboratory (LNLS) coupled to a Marmosaic-225 CCD detector (Mar, USA). The diffraction data were indexed, integrated and scaled using HKL2000 [73].

### X-ray Structure Determination and Refinement

The crystal structure of VirB7_XAC2622_51–134_ was determined by molecular replacement by Phaser [74] using residues 51–134 of the lowest energy NMR structure as the search model. The model was iteratively refined using the graphics program Coot [75] with rounds of restrained refinement in Refmac5 [76] with individual anisotropic *B*-factor for all non-hydrogen atoms. All programs used for refinement were from the CCP4 suite [77]. The quality of the structure was analyzed by the programs Coot, Refmac5, Procheck [78], Rampage [79] and MolProbity [80]. The coordinates of the crystal structure were deposited in the PDB (PDB code 3OV5).

### Fluorescence Spectroscopy

Fluorescence experiments were conducted in an AVIV ATF-105 spectrofluorimeter (AVIV Instruments). Fluorescence emission was collected after successive addition of 0.1 µM VirB7_XAC2622_24–139_His_ aliquots into a sample containing 1 µM VirB9_XAC2620_34–255_ in 5 mM sodium acetate (pH 5.0). The final VirB7_XAC2622_24–139_His_ concentration in the titration was 2.2 µM. Samples were pre-equilibrated for 2 min at 25°C, excited at 280 nm (2 nm bandwidth) and fluorescence emission was detected from 334–342 nm (7 nm bandwidth) at 2 nm intervals. The dissociation constant was calculated from a nonlinear regression fit of fluorescence titration data to Eq. 2 [81], [82], using the SigmaPlot program (SPSS, Inc.):
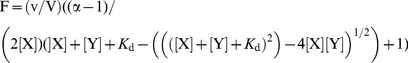
(2)where F is the fluorescence intensity at any given point of the titration curve, v is the initial volume, V is volume at any given point of the titration, [X] and [Y] are the VirB9_XAC2620_34–255_ and VirB7_XAC2622_24–139_His_ concentrations at any given point of the titration, respectively, *K*_d_ is the dissociation constant and α is the ratio between the maximum fluorescence intensity and the initial fluorescence intensity.

### Glutaraldehyde Cross-Linking Experiments

Samples with different concentrations of VirB7_XAC2622_24–139_ or VirB7_XAC2622_51–134_ were pre-equilibrated for 15 min, and then incubated with 0.01% (v/v) glutaraldehyde. After 20 min, the cross-linking reaction was stopped by the addition of SDS-PAGE sample buffer (100 mM Tris-HCl pH 6.8, 3.7% (w/v) SDS, 18.7% (v/v) glycerol, 140 mM 2-mercaptoethanol and 0.01% (w/v) bromophenol blue). The cross-linking products were analyzed by 16% Tricine SDS-PAGE [83].

### *virB7_XAC2622_* Knockout and Complementation

Approximately 1 kb of the upstream and downstream regions of the *virB7_XAC2622_* gene were amplified by PCR from Xac genomic DNA and the two fragments were ligated to produce an in frame deletion, leaving only the region coding for the first three and last five codons. This sequence was then cloned into the *Bam*HI restriction site of the pNPTS138 suicide vector (M. R. Alley, unpublished), thereby generating the plasmid pNPTS-Δ*xac2622*. This vector was introduced into Xac by electroporation and replacement of the wild-type copy by the deleted version was obtained after two recombination events as described [84]. In order to complement the *virB7_XAC2622_* knockout, a fragment including the *virB7_XAC2622_* gene plus 1000 pb of upstream sequence was amplified by PCR from Xac genomic DNA and inserted into the pUFR047 vector [85] at the *Bam*HI restriction site, creating the plasmid pUFR-VirB7. This plasmid was then transferred to the Δ*virB7_XAC2622_* strain by electroporation and selection of gentamicin resistance.

### *In planta* Studies

Virulence assays were performed by infiltration of the Xac strains into sweet orange leaves (*Citrus sinensis* L. Osbeck). The cell cultures were adjusted to an optical density of 0.1 at 600 nm and leaves were inoculated by syringe infiltration with needle. The plants were maintained at 28°C with a 12 h photoperiod and the development of the symptoms was regularly observed. Bacterial growth was measured by harvesting 10 mm^2^ citrus leaf discs for each inoculated Xac strain, and the leaf discs were macerated in 150 mM sodium chloride. The solution was serially diluted and spread onto LBON plates [1% (w/v) tryptone, 0.5% (w/v) yeast extract and 1.5% (w/v) agar] containing ampicillin. The mean number of colony forming units per square centimeter (CFU/cm^2^) was calculated by counting individual colonies obtained from each dilution.

### Antibody Production and Immunoblot

Rabbit polyclonal antibodies were raised against VirB7_XAC2622_24–139_, VirB9_XAC2620_34–255_ and VirB10_XAC2619_85–389_His_. To analyze protein levels in Xac, cells were grown in XVM2 medium [86] to an optical density (600 nm) of 1.0. Recombinant proteins and Xac cellular extracts were separated by 18% SDS-PAGE followed by immunoblot analysis. The rabbit sera were used at 1∶1000 dilutions and the antibodies were detected with staphylococcal protein A conjugated to horseradish peroxidase (Sigma). Immunoblots were developed with the ECL Advance Western Blotting system and exposed to Amersham Hyperfilm ECL film (GE Healthcare).

### Immunoprecipitation

Strains of Xac were grown in XVM2 to an optical density (600 nm) of 1.0 and equal numbers of cells were harvested by centrifugation. Cells were resuspended in 500 µl of 50 mM Hepes (pH 8.0) containing 5 mM EDTA. Coimmunoprecipitation experiments were performed after solubilization of cells with deoxycholate (DOC) and N,N–Dimethyldodecylamine N-oxide (LDAO) detergents, as described [87]. Bacterial suspensions were treated with 200 µg/ml lysozyme for 1 h at 4°C. Cells were lysed by sonication in the presence of 100 mM NaCl, 1% (w/v) DOC and protease inhibitor cocktail (Sigma Aldrich). Volumes were adjusted to 1 mL with 50 mM Hepes (pH 8.0) and 2% (w/v) LDAO and lysates were solubilized by incubation for 16 h at 4°C with agitation. Solubilized material was isolated by centrifugation at 15,000×g for 20 min and incubated with Protein G agarose beads (Millipore) for 4 h at 4°C, removing nonspecifically bound proteins. For immunoprecipitation, supernatants from this pre-clearing step were incubated with protein G agarose beads in the presence of pre-immune serum (controls) or anti-VirB7, anti-VirB9 or anti-VirB10 antibody, for 16 h at 4°C, with agitation. The unbound material was discarded after centrifugation (400×g, 5 min) and beads were washed three times with decreasing concentrations of LDAO and DOC (final wash contained 0.1% DOC and 0.1% LDAO), each time for 10 min at 25°C, with agitation. Immunoprecipitates were eluted by addition of SDS sample buffer and boiling for 10 min. Samples were analyzed by SDS-PAGE followed by immunoblot.

### Quantitative RT-PCR

RNAs from Xac cultures grown in XVM2 medium to an optical density (600 nm) of 1.0 were extracted using Illustra RNAspin Mini kit, according to manufacturer's instructions (GE Healthcare). Purified RNA was treated with 1 U/µg DNaseI, RNase-free (Fermentas) and successful removal of contaminating DNA was confirmed by PCR. Reverse transcription was performed using 1 µg of DNaseI-treated RNA and RevertAid H Minus First Strand cDNA Synthesis Kit, following manufacturer's protocol (Fermentas). Quantitative amplification of the resulting cDNA (40 ng) was performed using 0.3 µM of each primer (F_virB9_RT and R_virB9_RT; Table S1) and SYBR Green/ROX qPCR Master Mix in the ABI7300 Real-Time System (Applied Biosystems). Relative quantification of gene expression was performed using gene *xac1631* (that codes for subunit A of DNA gyrase) as an endogenous control and the 2^−ΔΔCT^ method [88]. Primers were designed using the PrimerExpress Software (Applied Biosystems). Triplicates of two independent biological samples were used.

### Accession Numbers

^1^H, ^13^C and ^15^N resonance assignments of VirB7_XAC2622_ were deposited in the BMRB: entry 17257. NMR and X-ray crystallography coordinates have been deposited in the PDB with accession codes 2L4W and 3OV5, respectively.

## Supporting Information

Figure S1 ^15^N-HSQC spectrum of ^15^N-VirB7_XAC2622_24–139_ at 7 µM, annotated with residue assignments. Side-chain ^1^H-^15^N resonances of asparagines, glutamines and tryptophans are also indicated. The histidine 39 peak (H39) is below the contour level in this picture and its position is indicated with a rectangle.
(TIF)

Figure S2 (A) VirB7_XAC2622_51–134_ crystals. (B) 2*Fo* - *Fc* (1.5 σ: blue) and *Fo* - *Fc* (3.0 σ: green and −3.0 σ: red) electron density maps.
(TIF)

Figure S3 The head-to-tail interaction of VirB7_XAC2622_. (A) ^15^N-HSQC spectra of ^15^N-labeled VirB7_XAC2622_51–134_ (200 µM) titrated with unlabeled VirB7_XAC2622_38–52_ (from 0 µM (cyan) to 740 µM (black)). Note that some signals suffer line-broadening during the titration and fall below the contour level in this picture. (B) Weighted chemical shift changes (Δδcomp) of VirB7_XAC2622_51–134_ observed upon addition of VirB7_XAC2622_38–52_. (C) Normalized weighted chemical shift changes (Δδcomp(norm)), comparing the Δδcomp due to VirB7_XAC2622_24–139_ oligomerization (yellow; data shown in Figure 2B) and the VirB7_XAC2622_51–134_ - VirB7_XAC2622_38–52_ interaction (blue; Figure S3B). The Δδcomp(norm) values were calculated by dividing the Δδcomp values for each residue by the Δδcomp value for residue I90 in each of the two experiments.
(TIF)

Figure S4 Glutaraldehyde cross-linking experiments of VirB7_XAC2622_24–139_ (A) and VirB7_XAC2622_51–134_ (B). (A) VirB7_XAC2622_24–139_ at 720 µM (lanes 1–3), 240 µM (lane 4), 80 µM (lane 5) and 27 µM (lane 6) were incubated without (lane 1) or with (lanes 2–6) 0.01% (v/v) glutaraldehyde. The incubation in lane 2 also contained 1% (w/v) SDS. (B) VirB7_XAC2622_24–139_ at 720 µM without (lane 1) or with 0.01% (v/v) glutaraldehyde (lane 2); VirB7_XAC2622_51–134_ at 720 µM (lanes 3 and 4), 240 µM (lane 5) and 80 µM (lane 6) were incubated without (lane 3) or with 0.01% (v/v) glutaraldehyde (lanes 4–6). Although the reactions were performed with different protein concentrations, the same amount of VirB7_XAC2622_24–139_ or VirB7_XAC2622_51–134_ was loaded in each lane of the 16% Tricine SDS-PAGE gel. MW: molecular weight marker.
(TIF)

Figure S5 VirB7_XAC2622_24–139_His_-VirB9_XAC2620_34–255_ interaction studied by fluorescence. X-axis: concentration of VirB7_XAC2622_24–139_His_. Y-axis: arbitrary fluorescence increase due to the interaction (F/Fo: Intensity/Initial intensity). Black dots: experimental data. White dots: fitted curve model for an interaction with a dissociation constant (*K*_d_) of approximately 4×10^−8^ M. VirB7_XAC2622_24–139_His_ has two tryptophans (W34 and W71) while VirB9_XAC2620_34–255_ has one (W177). See Materials and Methods for experimental details.
(TIF)

Figure S6 ^1^H-^15^N residual dipolar couplings (RDCs) of VirB7_XAC2622_24–139_. Blue: ^15^N- VirB7_XAC2622_24–139_ alone; red: ^15^N- VirB7_XAC2622_24–139_ in complex with ^14^N-VirB9_XAC2620_154–255_. The N-terminal region with largest RDC value differences corresponds to the VirB9_XAC2620_154–255_ binding site in VirB7_XAC2622_24–139_. The VirB7_XAC2622_24–139_ concentration was 250 µM in both experiments, with the addition of a 40% excess of VirB9_XAC2620_154–255_ in the case of the VirB7-VirB9 complex.
(TIF)

Figure S7 The interaction between VirB7_XAC2622_24–139_ and VirB9_XAC2620_154–255_ does not affect the oligomerization of VirB7_XAC2622_24–139_. The cross-peak positions of the same ^15^N-VirB7_XAC2622_24–139_ residues shift in fast exchange upon dilution both in the absence (left) and in the presence (right) of ^14^N-VirB9_XAC2620_154–255_. VirB7_XAC2622_24–139_ or VirB7_XAC2622_24–139_-VirB9_XAC2620_154–255_ complex concentrations: 800 µM (cyan), 400 µM (green) and 200 µM (orange). The arrows indicate protein dilutions from 800 to 200 µM.
(TIF)

Figure S8 The conformation of the VirB9_XAC2620_ C-terminal domain changes significantly upon interacting with VirB7_XAC2622_. ^15^N-HSQC spectra of ^15^N-VirB9_XAC2620_154–255_ in the absence (red) and in the presence (green) of ^14^N-VirB7_XAC2622_24–46_. Spectra were collected at 40°C on a 600 MHz spectrometer equipped with a cold probe.
(TIF)

Table S1 Oligonucleotides, plasmids and strains used in this study.
(DOC)
