# Supplementary figures and images for: A Component of the Xanthomonadaceae Type IV Secretion System Combines a VirB7 Motif with a N0 Domain Found in Outer Membrane Transport Proteins
Source: PLoS Pathog. 2011 May 12;7(5):e1002031. doi: 10.1371/journal.ppat.1002031 (PMC3093366; doi:10.1371/journal.ppat.1002031)

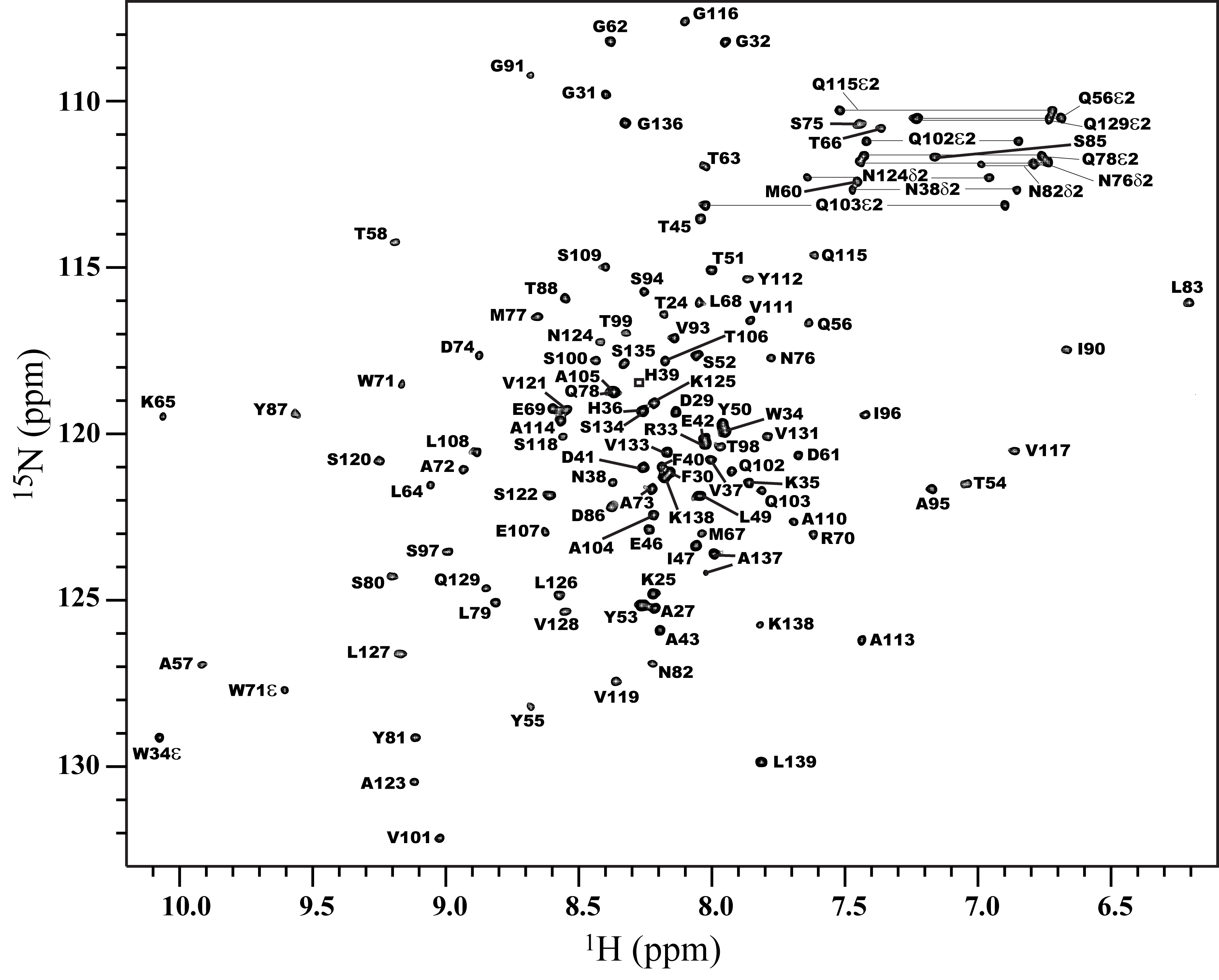

Supplement: Figure S1 — 15N-HSQC spectrum of 15N-VirB7XAC2622_24–139 at 7 µM, annotated with residue assignments. Side-chain 1H-15N resonances of asparagines, glutamines and tryptophans are also indicated. The histidine 39 peak (H39) is below the contour level in this picture and its position is indicated with a rectangle. (TIF) [file ppat.1002031.s001.tif]

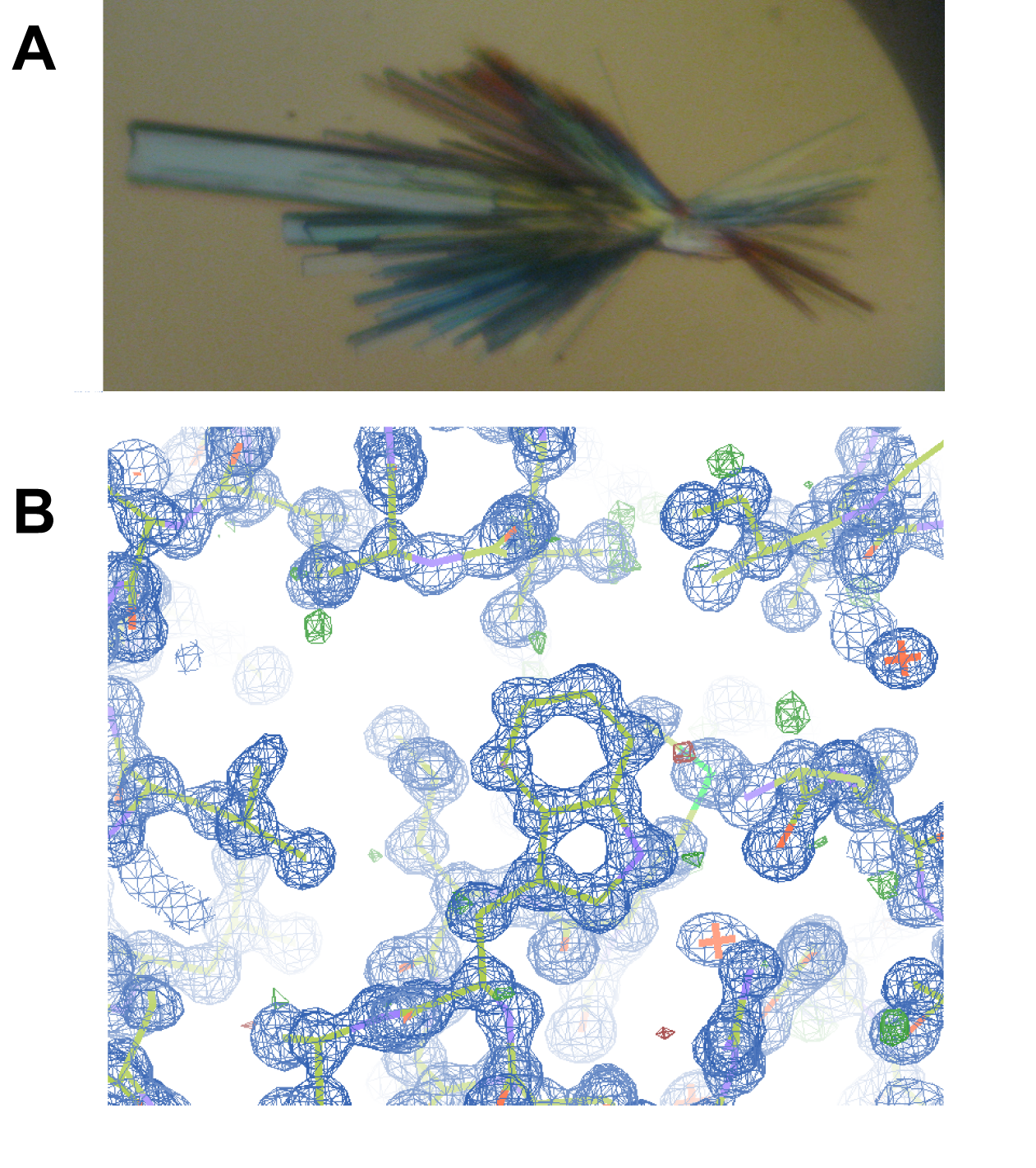

Supplement: Figure S2 — (A) VirB7XAC2622_51–134 crystals. (B) 2Fo - Fc (1.5 σ: blue) and Fo - Fc (3.0 σ: green and −3.0 σ: red) electron density maps. (TIF) [file ppat.1002031.s002.tif]

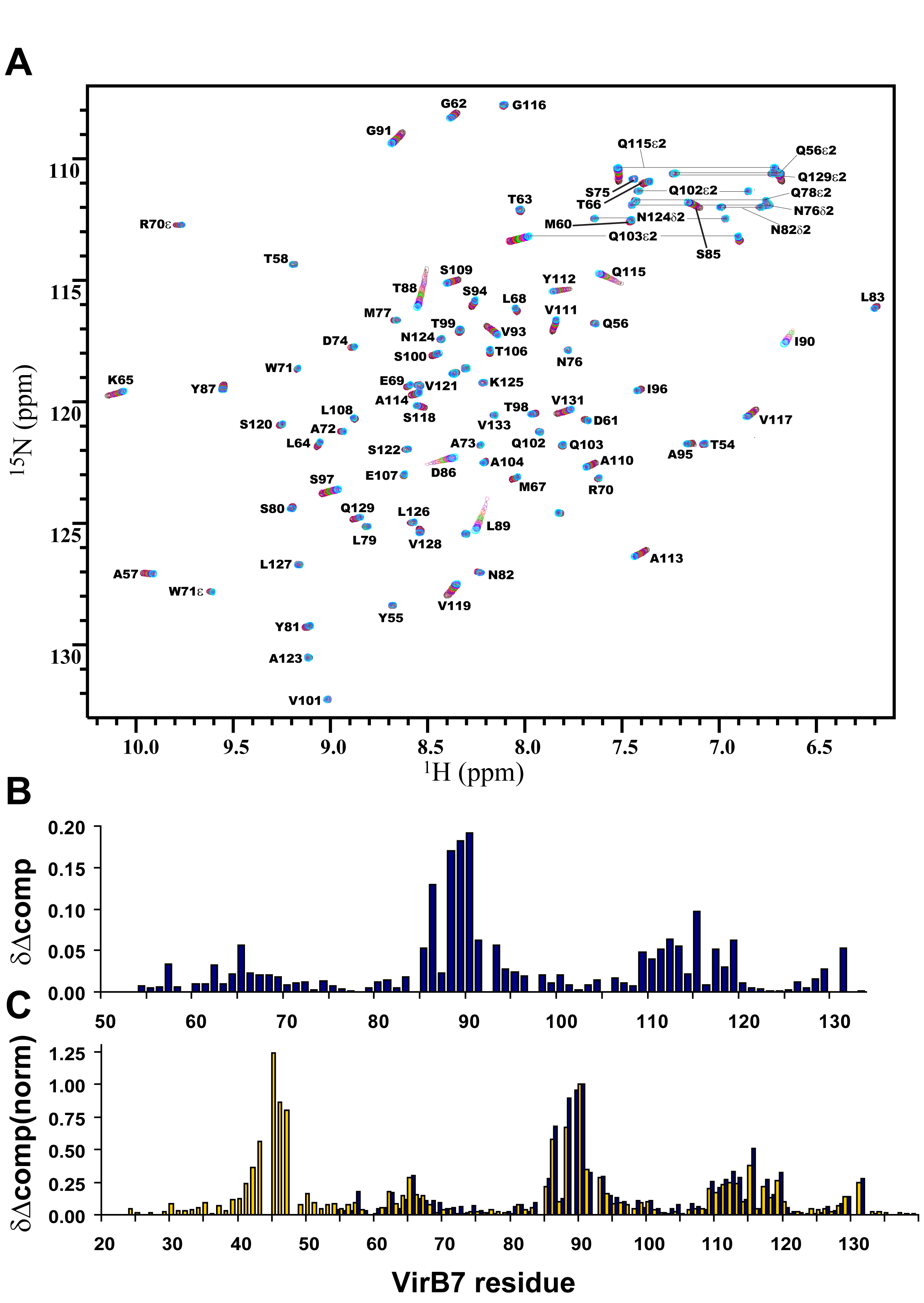

Supplement: Figure S3 — The head-to-tail interaction of VirB7XAC2622. (A) 15N-HSQC spectra of 15N-labeled VirB7XAC2622_51–134 (200 µM) titrated with unlabeled VirB7XAC2622_38–52 (from 0 µM (cyan) to 740 µM (black)). Note that some signals suffer line-broadening during the titration and fall below the contour level in this picture. (B) Weighted chemical shift changes (Δδcomp) of VirB7XAC2622_51–134 observed upon addition of VirB7XAC2622_38–52. (C) Normalized weighted chemical shift changes (Δδcomp(norm)), comparing the Δδcomp due to VirB7XAC2622_24–139 oligomerization (yellow; data shown in Figure 2B) and the VirB7XAC2622_51–134 - VirB7XAC2622_38–52 interaction (blue; Figure S3B). The Δδcomp(norm) values were calculated by dividing the Δδcomp values for each residue by the Δδcomp value for residue I90 in each of the two experiments. (TIF) [file ppat.1002031.s003.tif]

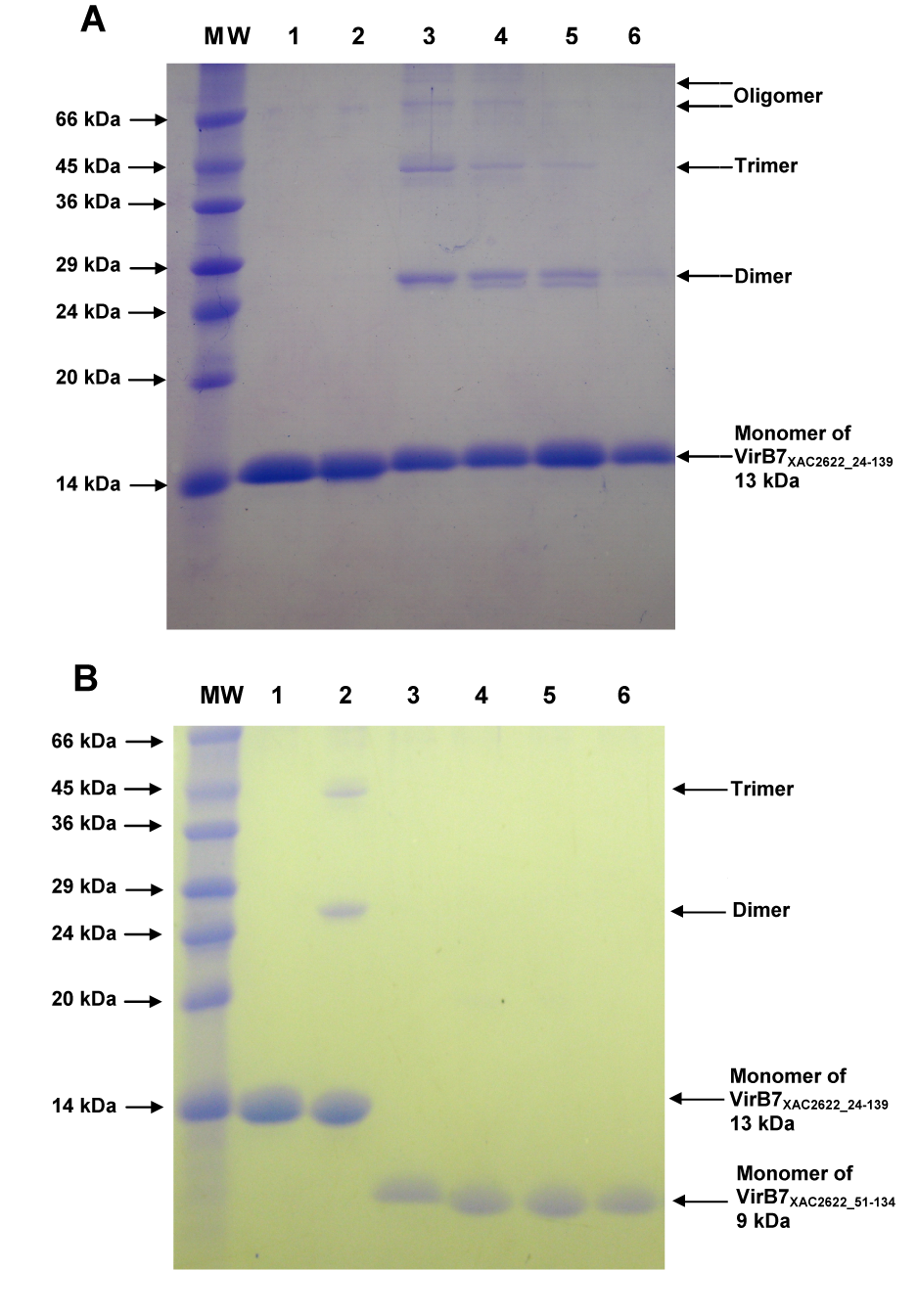

Supplement: Figure S4 — Glutaraldehyde cross-linking experiments of VirB7XAC2622_24–139 (A) and VirB7XAC2622_51–134 (B). (A) VirB7XAC2622_24–139 at 720 µM (lanes 1–3), 240 µM (lane 4), 80 µM (lane 5) and 27 µM (lane 6) were incubated without (lane 1) or with (lanes 2–6) 0.01% (v/v) glutaraldehyde. The incubation in lane 2 also contained 1% (w/v) SDS. (B) VirB7XAC2622_24–139 at 720 µM without (lane 1) or with 0.01% (v/v) glutaraldehyde (lane 2); VirB7XAC2622_51–134 at 720 µM (lanes 3 and 4), 240 µM (lane 5) and 80 µM (lane 6) were incubated without (lane 3) or with 0.01% (v/v) glutaraldehyde (lanes 4–6). Although the reactions were performed with different protein concentrations, the same amount of VirB7XAC2622_24–139 or VirB7XAC2622_51–134 was loaded in each lane of the 16% Tricine SDS-PAGE gel. MW: molecular weight marker. (TIF) [file ppat.1002031.s004.tif]

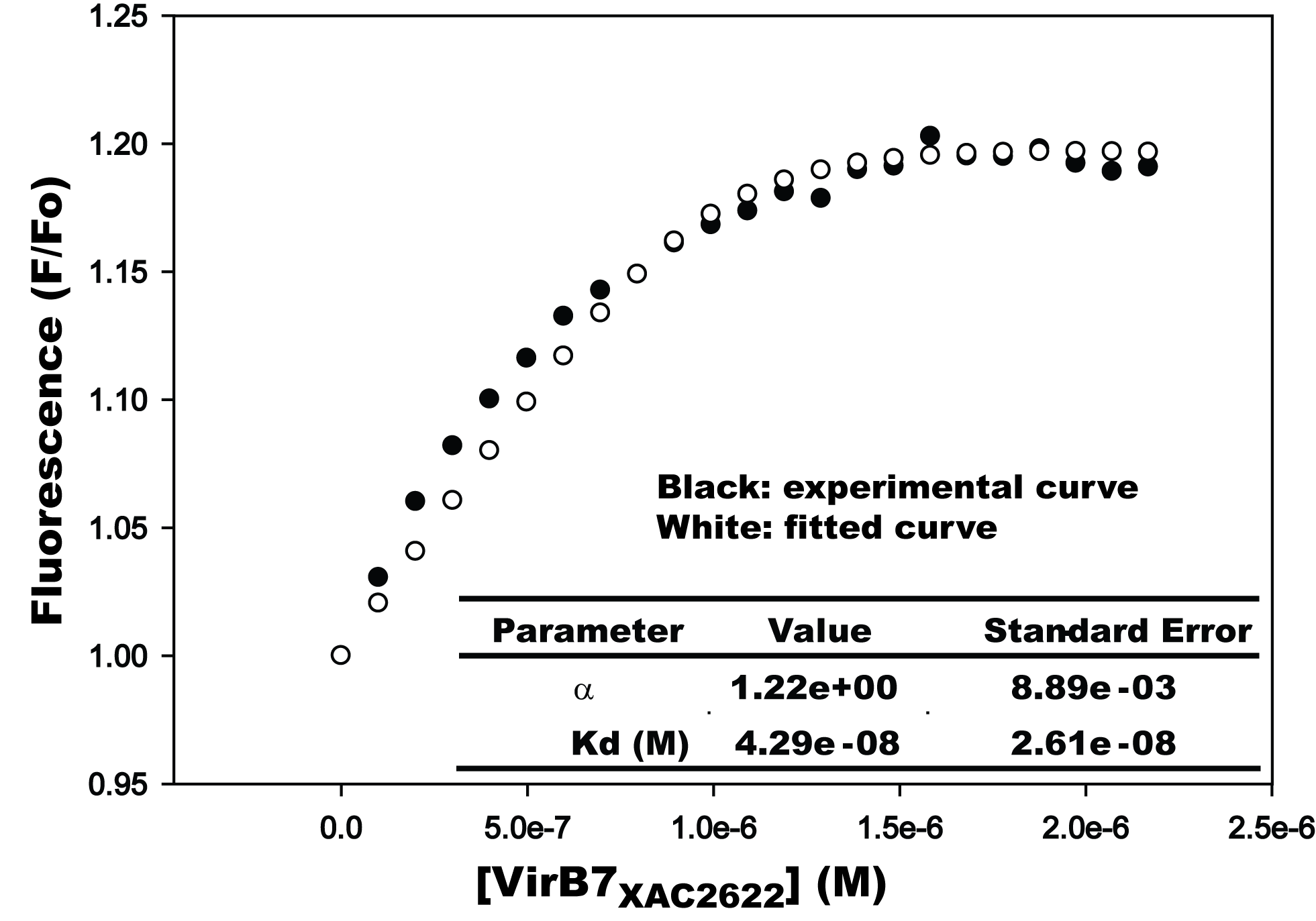

Supplement: Figure S5 — VirB7XAC2622_24–139_His-VirB9XAC2620_34–255 interaction studied by fluorescence. X-axis: concentration of VirB7XAC2622_24–139_His. Y-axis: arbitrary fluorescence increase due to the interaction (F/Fo: Intensity/Initial intensity). Black dots: experimental data. White dots: fitted curve model for an interaction with a dissociation constant (K d) of approximately 4×10^−8^ M. VirB7XAC2622_24–139_His has two tryptophans (W34 and W71) while VirB9XAC2620_34–255 has one (W177). See Materials and Methods for experimental details. (TIF) [file ppat.1002031.s005.tif]

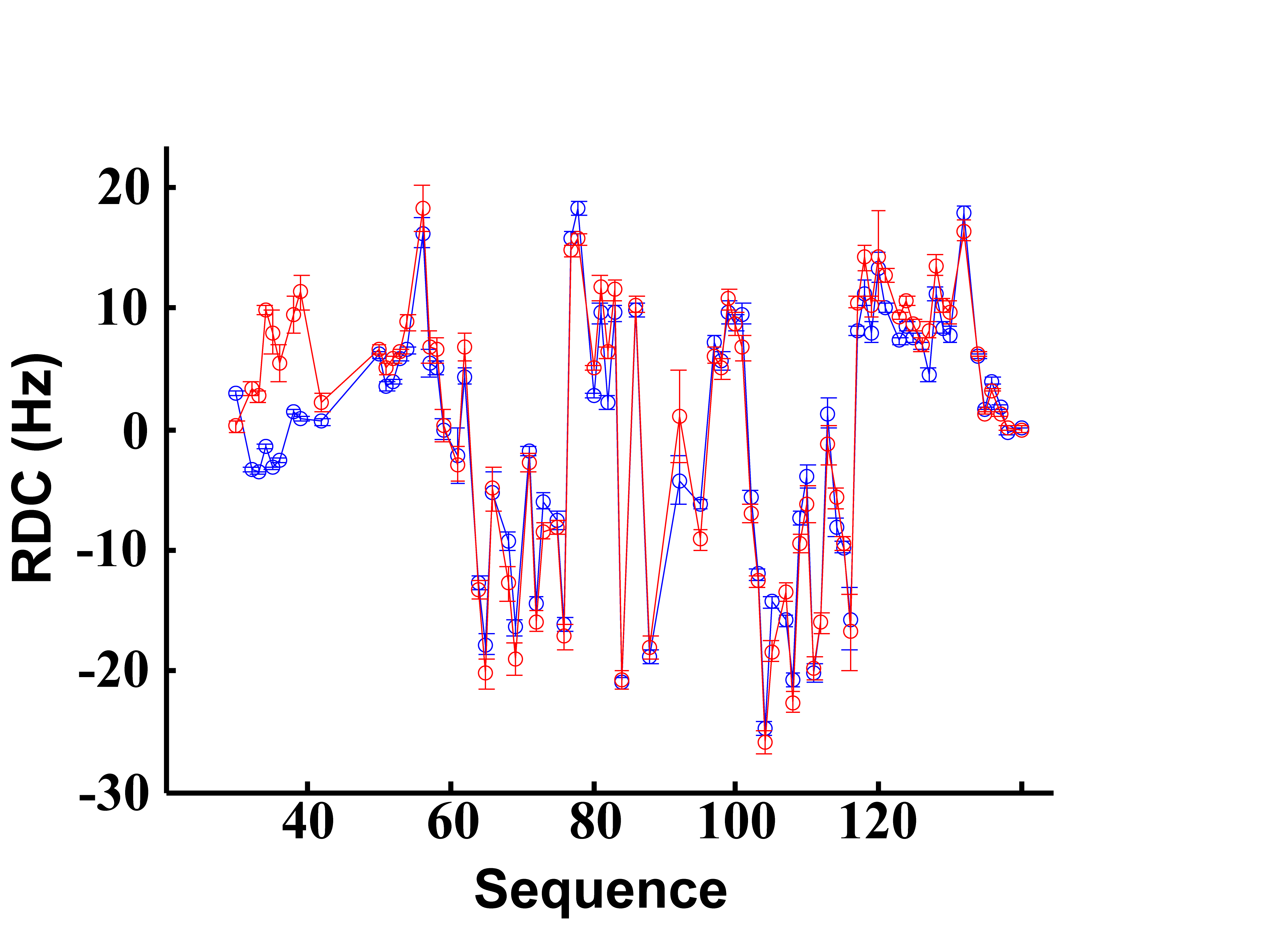

Supplement: Figure S6 — 1H-15N residual dipolar couplings (RDCs) of VirB7XAC2622_24–139. Blue: 15N- VirB7XAC2622_24–139 alone; red: 15N- VirB7XAC2622_24–139 in complex with 14N-VirB9XAC2620_154–255. The N-terminal region with largest RDC value differences corresponds to the VirB9XAC2620_154–255 binding site in VirB7XAC2622_24–139. The VirB7XAC2622_24–139 concentration was 250 µM in both experiments, with the addition of a 40% excess of VirB9XAC2620_154–255 in the case of the VirB7-VirB9 complex. (TIF) [file ppat.1002031.s006.tif]

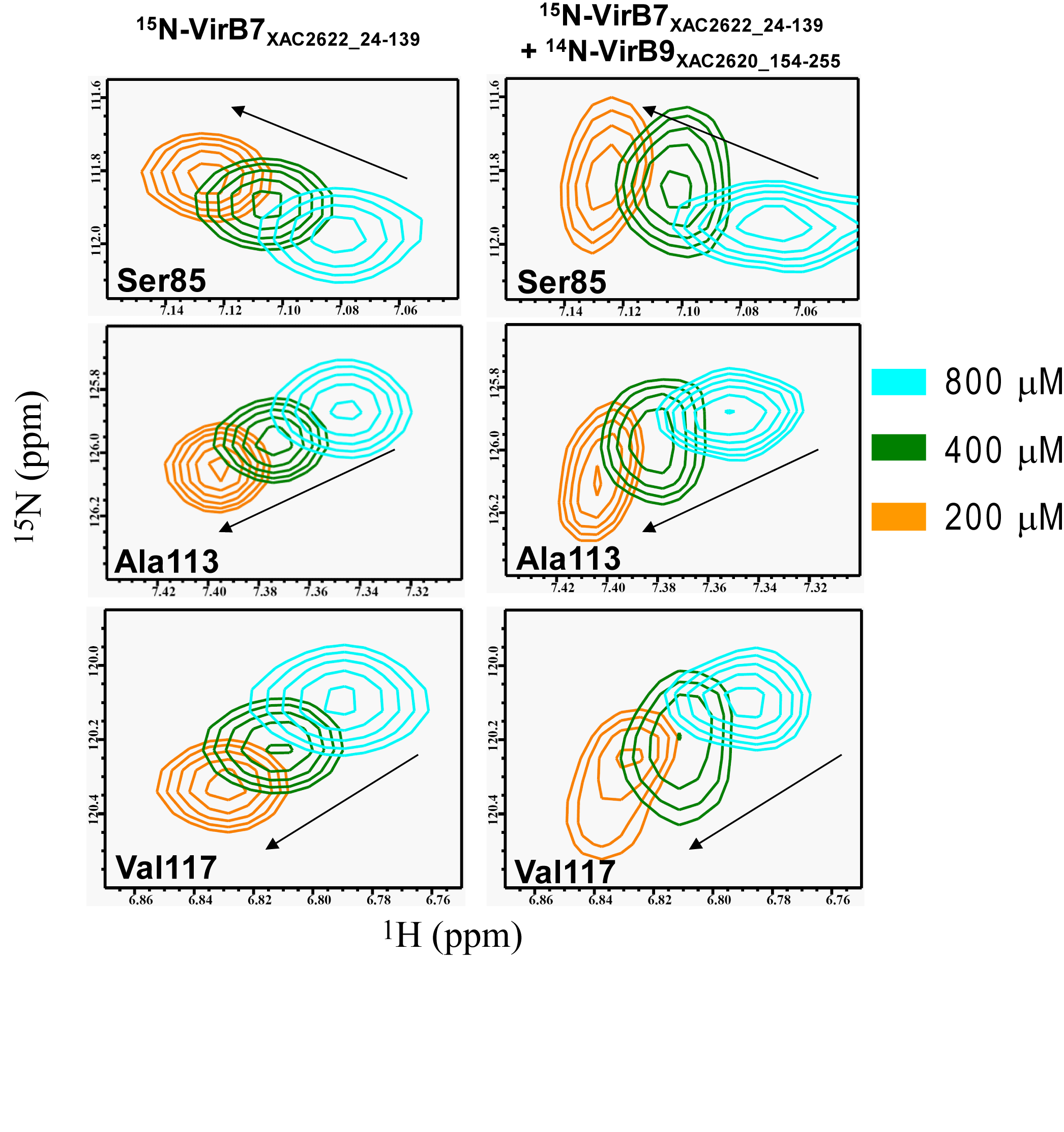

Supplement: Figure S7 — The interaction between VirB7XAC2622_24–139 and VirB9XAC2620_154–255 does not affect the oligomerization of VirB7XAC2622_24–139. The cross-peak positions of the same 15N-VirB7XAC2622_24–139 residues shift in fast exchange upon dilution both in the absence (left) and in the presence (right) of 14N-VirB9XAC2620_154–255. VirB7XAC2622_24–139 or VirB7XAC2622_24–139-VirB9XAC2620_154–255 complex concentrations: 800 µM (cyan), 400 µM (green) and 200 µM (orange). The arrows indicate protein dilutions from 800 to 200 µM. (TIF) [file ppat.1002031.s007.tif]

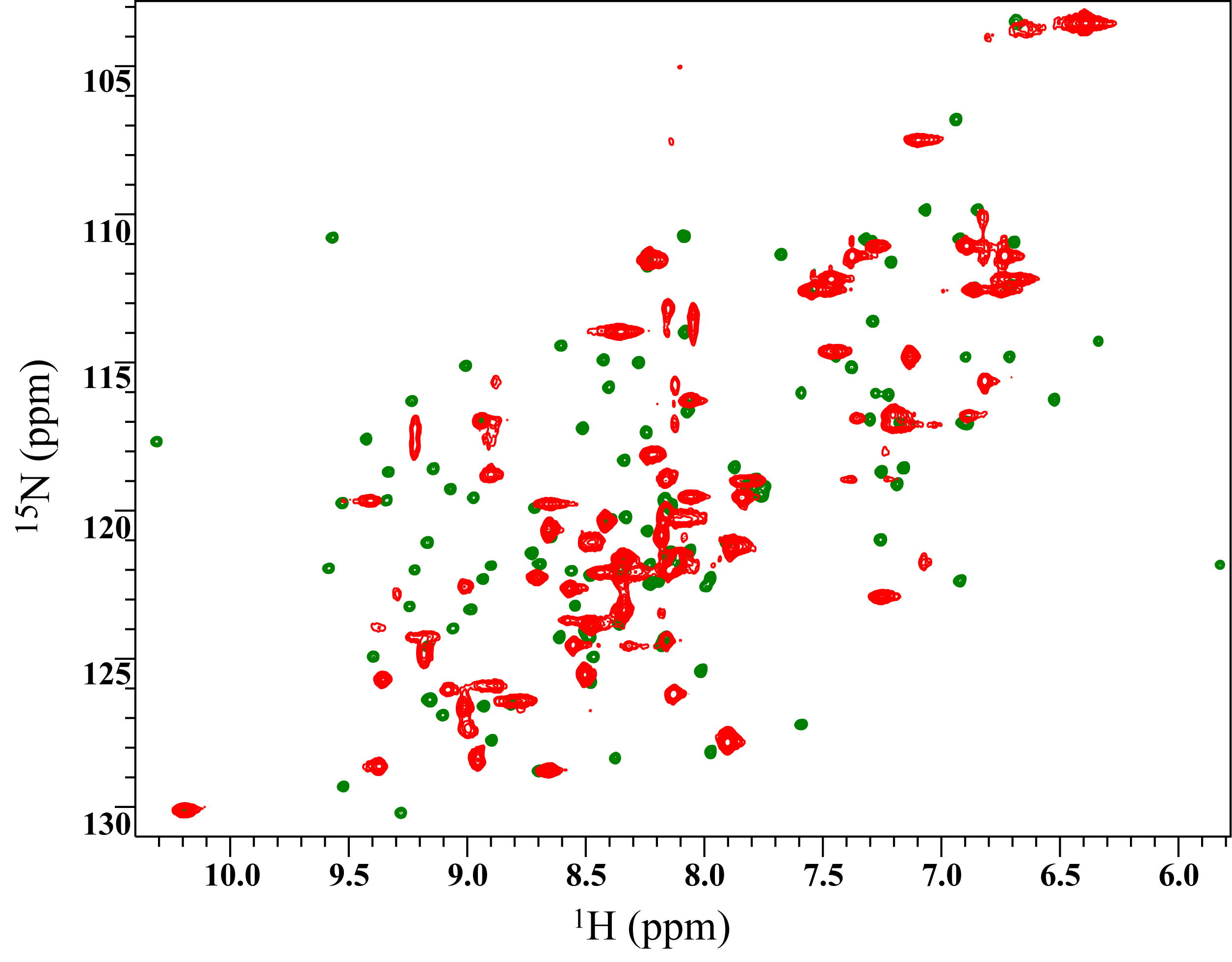

Supplement: Figure S8 — The conformation of the VirB9XAC2620 C-terminal domain changes significantly upon interacting with VirB7XAC2622. 15N-HSQC spectra of 15N-VirB9XAC2620_154–255 in the absence (red) and in the presence (green) of 14N-VirB7XAC2622_24–46. Spectra were collected at 40°C on a 600 MHz spectrometer equipped with a cold probe. (TIF) [file ppat.1002031.s008.tif]
