# Supplementary material for: A Component of the Xanthomonadaceae Type IV Secretion System Combines a VirB7 Motif with a N0 Domain Found in Outer Membrane Transport Proteins
Source: PLoS Pathog. 2011 May 12;7(5):e1002031. doi: 10.1371/journal.ppat.1002031 (PMC3093366; doi:10.1371/journal.ppat.1002031)
Supplement: Table S1 — Oligonucleotides, plasmids and strains used in this study. (DOC) [file ppat.1002031.s009.doc]

**Table S1.** Oligonucleotides, plasmids and strains used in this study.

| **Oligonucleotides** | **Description** | **Sequence** |
| --- | --- | --- |
| F-VirB7XAC2622_24 | Forward primer for cloning of *virB7_XAC2622_* from codon 24 | CCTGAGACATATgaccaagcctgctcctgac |
| F-VirB7XAC2622_51 | Forward primer for cloning of *virB7_XAC2622_* from codon 51 | cgaattccatatgacctcgtacacgtatcaggc |
| R-VirB7XAC2622_134 | Reverse primer for cloning of *virB7_XAC2622_* until codon 134 | gaggatccttAcgacaccggcactggttg |
| R-VirB7XAC2622_139 | Reverse primer for cloning of *virB7_XAC2622_* until codon 139 | TTTAAGCTTCTCGAGTTAGAGCTTGGCGCCAGACG |
| F-VirB9XAC2620_34 | Forward primer for cloning of *virB9_XAC2620_* from codon 34 | GCGGAATTCCATATGTATGCACCCGATCGTATCTATC |
| F-VirB9XAC2620_154 | Forward primer for cloning of *virB9_XAC2620_* from codon 154 | gccgtgcCataTGAATGCCAAGATCTTGAAGGATC |
| R-VirB9XAC2620_255 | Reverse primer for cloning of *virB9_XAC2620_* until codon 255 | AACTGCAGGATCCTTACTTTTGCTTGTTCCTTCGCAGAC |
| F-VirB10XAC2619_85 | Forward primer for cloning of *virB10_XAC2619_* from codon 85 | GACTAGCCATATGCAAAGCGGGGAGGATTCC |
| R-VirB10XAC2619_389 | Reverse primer for cloning of *virB10_XAC2619_* until codon 389 | TAGGATCCTCGAGTCACTTCGGCAGGACAGC |
| F-VirB7XAC2622_U | Forward primer for cloning of 1 kb upstream of *virB7_XAC2622_* | TTCTGGATCCGCTGCAACCAGTGCGGACG |
| R-VirB7XAC2622_U | Reverse primer for cloning of 1 kb upstream of *virB7_XAC2622_* | TACATCCATGGATATTCACTCTGACTTCTCCAACTCATG |
| F-VirB7XAC2622_D | Forward primer for cloning of 1 kb downstream of *virB7_XAC2622_* | GTCGTCCATGGCGCCAAGCTCTAAGGGCTGC |
| R-VirB7XAC2622_D | Reverse primer for cloning of 1 kb downstream of *virB7_XAC2622_* | CATTTGGATCCGGCCATCCCAGGCATCG |
| F_VirB9_RT | Forward primer for qRT-PCR  for *virB9_XAC2620_* gene | TGAGCCCGAACGAAAAAATT |
| R_VirB9_RT | Reverse primer for qRT-PCR  for *virB9_XAC2620_* gene | AAAAAACATTCTCTCGCCGC |
| F_xac1631 | Forward primer for qRT-PCR  for *XAC1631* gene | CCTGTATCAGCAGACCCAGATG |
| R_xac1631 | Reverse primer for qRT-PCR  for *XAC1631* gene | GGATGAACGCCTCCAGCAT |

| **Plasmids** | **Properties** | **Source/reference** |
| --- | --- | --- |
| pET11a | *E. coli* expression vector | Novagen |
| pET28a | *E. coli* expression vector | Novagen |
| pET11a-VirB7XAC2622_51-134 | pET11a encoding VirB7XAC2622_51-134 | This study |
| pET11a-VirB9XAC2620_34-255 | pET11a encoding VirB9XAC2620_34-255 | This study |
| pET28a-VirB7XAC2622_24-139_His | pET28a encoding VirB7XAC2622_24-139_His | This study |
| pET28a-VirB9XAC2620_154-255_His | pET28a encoding VirB9XAC2620_154-255_His | This study |
| pET28a-VirB10XAC2619_85-389_His | pET28a encoding VirB10XAC2619_85-389_His | This study |
| pNPTS138 | Suicide vector for generation of gene knockouts (*sacB* Kan^r^) | M. R. Alley, unpublished |
| pNPTS-Δ*xac2622* | pNPTS138 containing 1 kb flanking regions of the upstream and downstream regions of the *xac2622* gene | This study |
| pUFR047 | Vector for gene complementation (Gm^r^) | [89] |
| pUFR-*VirB7* | pUFR047 for complementation of *virB7_XAC2622_* (including 1 kb of upstream sequence) | This study |

| **Strains** | **Genotype** | **Source/reference** |
| --- | --- | --- |
| BL21(DE3)RP | *E. coli* B strain carrying DE3 prophage and pACYC-based plasmids expressing rare tRNAs for Arg and Pro | Novagen |
| Xac | *Xanthomonas citri* subsp. citri strain 306 (Xac 306) | [14] |
| Δ*virB7_XAC2622_* | Xac 306 Δ*virB7_XAC2622_* | This study |
| Δ*virB7_XAC2622_* + p2622 | Δ*virB7_XAC2622_* containing vector pUFR-VirB7 | This study |
